# Distinct functions and transcriptional signatures in orally induced regulatory T cell populations

**DOI:** 10.3389/fimmu.2023.1278184

**Published:** 2023-10-26

**Authors:** Moanaro Biswas, Kaman So, Thais B. Bertolini, Preethi Krishnan, Jyoti Rana, Maite Muñoz-Melero, Farooq Syed, Sandeep R. P. Kumar, Hongyu Gao, Xiaoling Xuei, Cox Terhorst, Henry Daniell, Sha Cao, Roland W. Herzog

**Affiliations:** ^1^ Herman B Wells Center for Pediatric Research, Indiana University School of Medicine, Indianapolis, IN, United States; ^2^ Department of Biostatistics and Health Data Science and Center for Computational Biology and Bioinformatics, Indiana University School of Medicine, Indianapolis, IN, United States; ^3^ Department of Chemical and Biological Engineering, University of British Columbia, Vancouver, BC, Canada; ^4^ Center for Medical Genomics, Indiana University School of Medicine, Indianapolis, IN, United States; ^5^ Division of Immunology, Beth Israel Deaconess Medical Center (BIDMC), Harvard Medical School, Boston, MA, United States; ^6^ Department of Basic and Translational Sciences, School of Dental Medicine, University of Pennsylvania, Philadelphia, PA, United States

**Keywords:** FoxP3+ regulatory T cells, latency associated peptide (LAP), oral tolerance, antidrug antibodies (ADA), single cell RNA and transcriptome sequencing

## Abstract

Oral administration of antigen induces regulatory T cells (Treg) that can not only control local immune responses in the small intestine, but also traffic to the central immune system to deliver systemic suppression. Employing murine models of the inherited bleeding disorder hemophilia, we find that oral antigen administration induces three CD4+ Treg subsets, namely FoxP3+LAP-, FoxP3+LAP+, and FoxP3-LAP+. These T cells act in concert to suppress systemic antibody production induced by therapeutic protein administration. Whilst both FoxP3+LAP+ and FoxP3-LAP+ CD4+ T cells express membrane-bound TGF-β (latency associated peptide, LAP), phenotypic, functional, and single cell transcriptomic analyses reveal distinct characteristics in the two subsets. As judged by an increase in IL-2Rα and TCR signaling, elevated expression of co-inhibitory receptor molecules and upregulation of the TGFβ and IL-10 signaling pathways, FoxP3+LAP+ cells are an activated form of FoxP3+LAP- Treg. Whereas FoxP3-LAP+ cells express low levels of genes involved in TCR signaling or co-stimulation, engagement of the AP-1 complex members Jun/Fos and Atf3 is most prominent, consistent with potent IL-10 production. Single cell transcriptomic analysis further reveals that engagement of the Jun/Fos transcription factors is requisite for mediating TGFβ expression. This can occur via an Il2ra dependent or independent process in FoxP3+LAP+ or FoxP3-LAP+ cells respectively. Surprisingly, both FoxP3+LAP+ and FoxP3-LAP+ cells potently suppress and induce FoxP3 expression in CD4+ conventional T cells. In this process, FoxP3-LAP+ cells may themselves convert to FoxP3+ Treg. We conclude that orally induced suppression is dependent on multiple regulatory cell types with complementary and interconnected roles.

## Introduction

The immune system of the gut and associated lymphoid tissue has evolved to maintain tolerogenic symbiosis with food antigens and commensal bacteria, while still being able to mount protective immune responses against pathogens (1). This unique tolerogenic property can be harnessed by orally delivering antigen in a dose dependent manner to achieve specific tolerance to allergens, auto-antigens and therapeutic proteins (2, 3). This is illustrated by the recent FDA approval of Palforzia, which is an incremental oral immunotherapy to desensitize patients with peanut allergy and mitigate allergic reactions that may occur after accidental exposure (4). Oral antigen administration has also shown encouraging results in animal models of autoimmune and inflammatory diseases (5–9). We have previously shown that this concept of oral tolerance can also be adapted to prevent anti-drug antibody (ADA) formation against therapeutic proteins used in replacement therapies for genetic diseases (10). For instance, repeated oral delivery of bioencapsulated human coagulation factor VIII (hFVIII) or IX (hFIX) antigens suppressed ADAs against intravenously (IV) delivered therapeutic protein in animal models of the X-linked bleeding disorder hemophilia A (FVIII deficiency) or hemophilia B (FIX deficiency) respectively (11–16). Importantly, tolerance initially induced via the intestinal immune system resulted in systemic suppression to the IV delivered antigen. The spleen is the site of initiation for ADA development, which requires an orchestrated effort by several anatomically distinct antigen presenting cells that prime the differentiation of T follicular helper cells, thereby potentiating the germinal center response and ADA formation (17).

Several factors are likely responsible for the emergence of tolerogenic antigen-specific T cells in the gut environment, including presentation of antigen without optimal costimulation in the small intestine and mesenteric lymph node (MLN), which promotes the expression of FoxP3, immune modulatory cytokines and other molecules (2, 10, 18–20). While it is known that active suppression by regulatory T cells (Tregs) is an integral component of the oral tolerance mechanism, substantial gaps in knowledge remain about the identities and suppression mechanisms of the specific subsets of orally induced Treg. A FoxP3^-^ T cell population that plays a major role in oral tolerance are CD4^+^ T cells that are characterized by surface expression of LAP, which maintains TGFβ in a latent state (21). LAP^+^ cells have been identified in multiple studies of allergy or autoimmunity, where they are induced by inhaled or ingested antigen or CD3 antibody (22–25). These cells are robustly induced in the gut immune system (Peyer’s patches and MLN) but are also detectable in the blood and spleen (12, 13). TGFβ and in some cases, IL-10 are proposed to be key molecules in mediating the suppressive capacity of LAP^+^ cells, because treatment with neutralizing antibodies to LAP and IL-10 abrogates the disease-protective effects of these cells (24, 26). However, the underlying mechanisms driving the induction of this unique cell type are less well characterized. LAP can also be upregulated on FoxP3^+^ Tregs, where its upregulation correlates with an activated phenotype with enhanced suppressive properties (20, 27, 28). Additionally, LAP expression has been identified on γδ T cells, microglia, dendritic cells, B cells and in a subset of monocytic myeloid derived suppressor cells (MDSCs) (29–32).

In our studies with orally delivered plant cell encapsulated clotting factor, tolerance was mediated by the induction of FoxP3^+^ and FoxP3^-^LAP^+^ subsets of CD4^+^ T cells through enhanced secretion of IL-10 and TGFβ (11–13). Recently, we demonstrated that a short course of low dose oral anti-CD3 monoclonal antibody was effective in inhibiting ADA responses to FVIII. Suppression was accompanied by early induction of FoxP3^+^LAP, FoxP3^-^LAP^+^, and FoxP3^+^LAP^+^ populations of CD4^+^ T cells in the spleen and MLN (33). Therefore, we have shown in multiple studies and via different treatment strategies that oral tolerance is established by a heterogenous population of T cells with a regulatory phenotype. Nonetheless, we believe that plant cell based oral antigen delivery is particularly attractive for several reasons. In our design, plant cells provide bioencapsulation that protects from degradation of the antigen in the stomach, high levels of expression can be achieved in chloroplasts, and a transmucosal carrier facilitates translocation to the intestinal immune system and therefore Treg induction (34, 35). In addition, freeze dried plant cells can be stored at ambient temperature for extended periods of time, circumventing the need for cold chain. In general, production in whole plants and delivery of plant cells substantially lower manufacturing costs by avoiding tissue culture/bioreactors and antigen purification (34, 35). Finally, drugs with general immune suppressive properties are avoided, thereby increasing safety (36).

Whether orally induced Treg populations represent distinct phenotypes or intermediate sub-phenotypes reflecting different states of maturation, differentiation, and activation is unclear. We therefore set out to profile and more accurately delineate the constituent Treg types responsible for mediating oral tolerance to ADA formation in hemophilia mice. Single cell transcriptomic analyses combined with immunophenotyping and functional assays revealed FoxP3^-^LAP^+^ cells to be a distinct subset from FoxP3^+^ Treg, while FoxP3^+^LAP^+^ double positive cells most likely represent an activated form of FoxP3^+^LAP^-^ Treg. Distinct from FoxP3^+^ Treg (which were characterized by high expression of IL-2 receptor and co-inhibitory molecules such as CTLA4), FoxP3^-^LAP^+^ Treg show low expression of genes involved in T cell receptor (TCR) signaling or costimulation (except for high ICOS expression) and most potently produce IL-10 in response to stimulation. FoxP3^+^LAP^+^ cells show a typical FoxP3^+^ Treg gene expression profile but upregulate TCR and Stat5 signaling pathways and share certain features with FoxP3^-^LAP^+^ Treg, including high induction of ICOS and Jun/Fos pathways. Finally, we provide evidence that FoxP3^-^LAP^+^ not only potently suppress CD4^+^ conventional T cell (T_conv_) proliferation but convert T_conv_ to FoxP3^+^ Treg and themselves may convert to FoxP3^+^ Treg.

## Materials and methods

### Animals

Hemophilia B (HB) mice on the C3H/HeJ background with a targeted deletion of the F9 gene were used as published (37, 38). Hemophilia A (HA, BALB/c *F8*e16^−/−^) mice were on the BALB/c genetic background with a deletion in exon 16 of the *F8* gene as previously described (11, 17, 39). Both strains were bred at Indiana University. All animal experiments were performed as per the guidelines of the Institutional Animal Care and Use Committee (IACUC) at Indiana University. Male mice, 6 to 8 weeks of age were used for *in vivo* experiments. The specific number of mice used in each cohort is indicated in the figure legends.

### Oral delivery of hFVIII or hFIX expressing transplastomic lettuce

HB or HA mice received a mixture of lettuce plant cells transgenic for either hFIX or the heavy chain (HC) or C2 domains of hFVIII (1.5 μg), fused to the cholera toxin B (CTB) subunit, which were generated as described (12, 40) and grown in a cGMP hydroponic facility (Fraunhofer CMB, DE). The lyophilized mixture was resuspended in 200μl PBS/mouse and delivered via oral gavage 2X/week for 2 months. 1 month into feeding, mice additionally received weekly injections of 1 IU rhFIX (Benefix, Pfizer, NY) or B domain deleted (BDD)-rhFVIII (Xyntha, Pfizer, NY) for 4 weeks.

### Flow cytometry

Spleens were harvested at indicated timepoints, single cell suspensions prepared, and surface and intracellular antibody staining performed according to manufacturers’ instructions. For transcription factor staining, cells were first surface stained, permeabilized with Foxp3/Transcription Factor Staining Buffer Set (eBioscience, San Diego, CA), incubated with antibody for 30 min at 4°C in the dark and then washed again in permeabilization buffer. For intracellular cytokine staining, cells were stimulated with Cell Activation Cocktail (PMA/Ionomycin with Brefeldin A, Biolegend, San Diego, CA) in culture with RPMI 1640 (Gibco) for 4 h at 37°C with 5% CO2. After incubation, cells were fixed and permeabilized with Foxp3/Transcription Factor Staining Buffer Set. Flow cytometry data were acquired on either Attune NxT Flow Cytometer (Life Technologies, Grand Island, NY) or Cytek Aurora (Northern Lights, Fremont, CA). All data were analyzed with FCS Express 7 (*De Novo* Software, Los Angeles, CA).

### Ex vivo co-culture

CD4^+^ T cells were magnetically enriched and labeled with PE conjugated LAP antibody (clone TW7-16B4, Biolegend). PE labeled cells were magnetically isolated using α-PE microbeads (Miltenyi Biotec) and further separated into (i) CD25^+^LAP^+^ (FoxP3^+^LAP^+^) and (ii) CD25^-^LAP^+^ (FoxP3^-^LAP^+^) cells by cell sorting (FACS Aria II). (iii) CD4^+^CD25^+^ cells were magnetically enriched using the mouse CD4^+^CD25^+^ regulatory T cell isolation kit (Miltenyi Biotec) and cell sorted for FoxP3^+^LAP^-^ cells. CD4^+^CD25^-^ T_conv_ cells were magnetically isolated and labeled with the CellTrace Violet dye (CTV, Invitrogen). CD4^+^FoxP3^-^LAP^-^ T_conv_ cells were co-cultured with (i) FoxP3^+^LAP^-^, (ii) FoxP3^+^LAP^+^, or (iii) FoxP3^-^LAP^+^ cells at a 2:1 ratio in a 48 well cell culture plate for 72hrs. CD4^+^ T cell depleted splenocytes were added as a source of antigen presenting cells at a 2:1 ratio of splenocytes: T_conv_ cells. Stimulation conditions included α-CD3/28 coated microbeads at a 1:1 ratio, soluble anti-mouse CD3 (1μg/ml), TGF-β (10ng/ml), or α-CD3/28 microbeads + TGFβ. After 72hrs, proliferation and FoxP3 induction in CTV labeled T_conv_ cells and in non-CTV labeled FoxP3^-^LAP^+^ cells was estimated by flow cytometry (BD Fortessa) and analyzed using the FCS express 7 software (de Novo software).

### mRNA sequencing

4 spleens from the hFIX lettuce group were isolated, magnetically enriched for CD4+ T cells and cell sorted into 2 populations on a FACs Aria II using a 4-way purity parameter: CD25^+^LAP^-^ (FoxP3^+^LAP^-^), and CD25^-^LAP^+^ (FoxP3^-^LAP^+^). We used CD25 as a surrogate cell surface marker for FoxP3, as there is generally a good correlation between CD25 and FoxP3 expression in murine splenic Tregs (3). FoxP3^+^LAP^-^ and FoxP3^-^LAP^+^ cells from the 4 spleens were then pooled, in order to obtain sufficient mRNA for the FoxP3-LAP+ population. RNA was isolated using the RNeasy Micro Kit (Qiagen), with DNase treatment performed according to the manufacturer’s instructions. Total RNA was evaluated using an Agilent Bioanalyzer 2100 (RNA integrity number of 9.9 – 10). One nanogram of total RNA per sample were used for library preparation. cDNA was first synthesized using SMART-Seq v4 Ultra Low Input RNA Kit for Sequencing (Takara Clontech Laboratories, Inc). Dual indexed cDNA library was then prepared using Nextera XT DNA Library Prep Kit (Illumina, Inc, San Diego, CA). Each library was quantified, and its quality accessed by Qubit and Agilent Bioanalyzer, and multiple libraries were pooled in equal molarity. Average size of library insert was about 300-400bp. The pooled libraries were then denatured, neutralized, before loading to NovaSeq 6000 sequencer for 100b paired-end sequencing (Illumina, Inc, San Diego, CA). Approximately 30M-40M reads per library was generated. Sequencing data were mapped by STAR RNA seq aligner, high quality reads were selected from BamUtils stats, mapped on gene from featureCounts and aligned to the mouse reference genome mm10. Ribosomal RNA reads were removed by Bowtie 2 (41). A Phred quality score (Q score) was used to measure the quality of sequencing. Q30 was 93%. 2 independent experiments of pooled samples (n=4/group) were run and combined. Data was normalized and batch effects were removed to generate a final dataset. Datasets were normalized using the fragments per kilobase per million mapped reads (FPKM) method.

DESeq2 package was used for normalization and differential expression (DE) analysis. Cutoff for differential expression was log2 Fold change >= 1.0 (linear scale-2.0), and a false discovery rate (FDR) <= 0.05 (Benjamini-Hochberg method). Corrected P values (<0.05) were taken as the threshold for significant differences in gene expression.

Identification and visualization of functional profiles for genes and gene clusters was performed using Database for Annotation, Visualization and Integrated Discovery (DAVID). Cutoff for enrichment analysis was fold change (linear scale) = 1.5 and p-value < 0.05. Significant gene ontology (GO) terms, including pathways, biological processes, cellular components, and molecular functions were identified from the enriched genes in the LAP^+^ dataset in relation to the FoxP3+ dataset. Terms with 30% or more genes in common were merged.​ Heatmaps were generated using the pheatmap package in R.

### Single cell library prep and sequencing

4 spleens from hFVIII lettuce treated animals were isolated, magnetically enriched for CD4^+^ T cells and sorted into CD4^+^CD25^+^, and CD4^+^CD25^-^LAP^+^ (FoxP3^-^LAP^+^) cells using the 4-way purity parameter on a FACS Aria II instrument. Cell debris, dead cells and aggregates were removed by filtration and low speed centrifugation, and cell number, viability and cell size were determined using a Countess II automated cell counter (Thermo Fisher Scientific, Waltham, MA). Approximately 10,000 targeted cell recovery per sample was applied to a single cell master mix with lysis buffer and reverse transcription reagents, following the Chromium NextGEM Single Cell 3’ Reagent Kits User Guide, CG000204 Rev D (10X Genomics, Inc, Pleasanton, CA). Along with the single cell gel beads and partitioning oil, the single cell master mixture containing the single cell suspension was dispensed onto a Single Cell Chip G in separate wells, and the chip loaded to the Chromium Controller for GEM generation and barcoding, followed by cDNA synthesis and library preparation. At each step, the quality of cDNA and library was examined by Bioanalyzer and Qubit. The resulting barcoded libraries were pooled in equal molarity and sequenced in a custom program for 28bp plus 91bp paired-end sequencing on Illumina NovaSeq 6000 (Illumina, Inc). Approximately 50K reads per cell were generated. Two sets of FoxP3^+^ and LAP^+^FoxP3^-^ cells were analyzed.

### Single cell analysis

CellRanger 5.0.1 (http://support.10xgenomics.com/) was implemented to demultiplex raw base sequence files into sample specific FASTQ files, which were then aligned to the mouse reference genome mm10 using RNAseq aligner STAR. Aligned reads were traced to individual cells and gene expression levels were quantified based on the number of UMIs detected. The filtered barcode matrices were used for further analysis. SoupX 1.5.2 was used to remove ambient RNA (42). Low quality cells were excluded based on the following criteria: unique features/gene counts >2500<500, >10% reads mapped to the mitochondrial genome.

Single cell RNA-seq data was analyzed in R4.1.2 using the Seurat package (v.4.2.0) (43–46). Samples were integrated using the function “IntegrateData” with 40 dimensions in the anchor weighting procedure in Seurat. We then selected variable genes and performed dimensionality reduction using principal component analysis and cell clustering on the integrated data with top 20 principal components using the “FindClusters” function in Seurat. Clusters are visualized by using the “RunUMAP” function with default setting except for setting the number of dimensions, i.e., dims, to be 20. Cluster specific markers were identified by performing differential gene expression using Wilcoxon test with a log fold change threshold of 0.25. We detected and annotated 3 unique cell types from the two samples. Differential expression analysis was performed between: (i) FoxP3^-^LAP^+^ and FoxP3^+^LAP^-^, (ii) FoxP3^-^LAP^+^ and FoxP3^+^LAP^+^, (iii) FoxP3^+^LAP^+^ and FoxP3^+^LAP^-^ populations using the DESeq2 package. A log2Fold change cutoff of 0.25, p value < 0.05 was used to consider a gene to be differentially expressed. The average cell yields from the three populations were 7314, 2353, 2349, respectively. This number of cells would yield at least 80% power to detect a log2 fold change of 0.25 using a two-sided Wilcoxon test with an FDR of 0.05. The power calculation is conducted using PASS Sample Size Software (NCSS LLC, Version 2019).

Pathway enrichment analysis was performed using the Molecular Signatures Database (MSigDB) vision 6, c2 canonical gene sets and c5 ontology gene sets (p-value < 0.05).

### Statistical analysis

Normality distribution for parametric tests was determined by the Shapiro-Wilk test. One-sided paired t-tests, and one-way ANOVA were employed, as specified in figure legends. For ANOVA, statistical significance was calculated by Tukey’s, Sidak’s or Dunnett’s *post hoc* tests. The level of significance was set at 0.05 for all tests. Statistical values for DEGs in both the mRNA seq and Single cell RNA-seq data were assessed using the built in statistical analysis tool in the DESeq2 package, which is based on the Wald test.

## Results

### Oral delivery of hFVIII induces multiple CD4^+^ T populations with a regulatory phenotype

**Figure 1:**
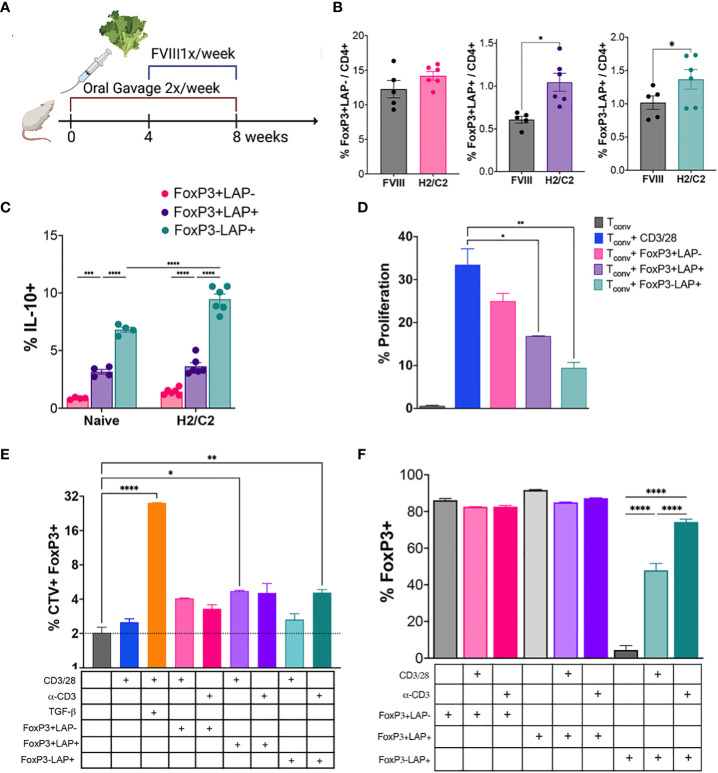
Oral gavage with hFVIII plant induces suppressive regulatory T cell populations. **(A)** Timeline for treatment. HA mice (n=5-6/group) orally received hFVIII HC/C2 expressing lyophilized lettuce reconstituted in PBS, administered 2X/week for 8 weeks. From weeks 4-8, animals additionally received weekly IV injections of rhFVIII. **(B)** Quantification of FoxP3^+^LAP^-^, FoxP3^+^LAP^+^, and FoxP3^-^LAP^+^ populations from CD4^+^ T cells of HA mice that received rhFVIII injections only, or rhFVIII injections combined with oral FVIII lettuce (HC/C2) treatment. **(C)** *In vitro* IL-10 production by FoxP3^+^LAP^-^, FoxP3^+^LAP^+^, and FoxP3^-^LAP^+^ populations from naïve or hFVIII HC/C2 plant fed animals. **(D)** Proliferation of CellTrace Violet labeled T_conv_ cells stimulated with αCD3/28 microbeads and co-cultured with FoxP3^+^LAP^-^, FoxP3^+^LAP^+^, and FoxP3^-^LAP^+^ cells. **(E)** Determination of FoxP3 induction in CellTrace Violet labeled T_conv_ cells stimulated with soluble αCD3 or αCD3/28 microbeads either supplemented with soluble TGFβ or co-cultured with FoxP3^+^LAP^-^, FoxP3^+^LAP^+^, or FoxP3^-^LAP^+^ cells. **(F)** FoxP3 expression in FoxP3^+^LAP^-^, FoxP3^+^LAP^+^, or FoxP3^-^LAP^+^ cells stimulated with soluble αCD3 or αCD3/28 microbeads and co-cultured with CellTrace Violet labeled T_conv_ cells. Statistical significance was calculated by paired T-test for **(B)**; one way ANOVA with Tukey’s *post hoc* test for **(C)**; one way ANOVA with Dunnett’s multiple comparisons test for **(D)** using αCD3/28 stimulated T_conv_ cells as control; one way ANOVA with Sidak’s multiple comparisons test for **(E)**, using unstimulated T_conv_ cells as control; one way ANOVA with Tukey’s multiple comparisons test for FoxP3^-^LAP^+^ co-culture. *p ≤ 0.05 **p ≤ 0.01; ***p ≤ 0.001; ****p ≤ 0.0001.

Previously, we established a protocol for suppression of inhibitor formation against IV injected rhFVIII by prior onset of oral delivery of bioencapsulated hFVIII antigen (11, 12). Freeze-dried lettuce leaf cells expressing CTB fusion proteins of the hFVIII heavy chain (HC) and C2 domains were repeatedly gavaged (2X/week, 1.5 μg per antigen) for 2 months. One month into oral gavage, weekly IV rhFVIII injections were administered for 4 weeks (**Figure 1A**). At the end of this period, we quantified frequencies of FoxP3^+^LAP^-^, FoxP3^+^LAP^+^ and FoxP3^-^LAP^+^ populations among CD4^+^ T cells in the spleens of fed and control animals that only received hFVIII injections (n=5/group, **Figure 1B**). Fluorescence minus one control was used to accurately determine the LAP^+^ population (**Supplementary Figure 1**). FVIII oral gavage significantly induced FoxP3^-^LAP^+^ T cells as compared to control hFVIII injected animals that did not receive oral gavage (1.72 vs 0.55% of CD4+, p=0.0011, paired t test) (**Figure 1B**). Frequencies of FoxP3^+^LAP^+^ Tregs were also increased in comparison to control animals (1.04 vs 0.60% of CD4^+^, p=0.0185, paired t test), whereas FoxP3^+^LAP^-^ Treg numbers were relatively unaffected (14.2% vs 12.27% of CD4^+^, **Figure 1B**).

An increased frequency of FoxP3^-^LAP^+^ T cells from hFVIII plant fed mice produced IL-10 in comparison to naive animals (9.46 vs 6.81%, p<0.0001, 2-way ANOVA), as quantified by intracellular cytokine staining (**Figure 1C**). The frequency of IL-10 expressing cells was significantly greater in FoxP3^-^LAP^+^ Tregs as compared to FoxP3^+^LAP^-^ Tregs under both fed and naïve conditions (p<0.0001, 2-way ANOVA). We also observed appreciable frequencies of IL-10 producing FoxP3^+^LAP^+^ Tregs in comparison to FoxP3^+^LAP^-^ Tregs under either treatment condition (p=0.0001 and <0.0001 respectively, **Figure 1C**).

### FoxP3^-^LAP^+^ cells show superior suppressive activity *in vitro*

TGFβ has been implicated in the conversion of CD4^+^FoxP3^-^ T_conv_ cells into CD4^+^FoxP3^+^ Tregs in response to TCR stimulation (47). We set up an *in vitro* co-culture assay in which CellTrace Violet (CTV) labeled CD4^+^ T_conv_ cells and T cell depleted splenocytes were incubated with sorted FoxP3^+^LAP^-^, FoxP3^+^LAP^+^, or FoxP3^-^LAP^+^ T cells. Cells were TCR stimulated with αCD3/28 coated microbeads or soluble αCD3 (clone 145-2C11), with or without TGFβ supplementation. First, we tested the *in vitro* suppressive capacity of FoxP3^+^LAP^-^, FoxP3^+^LAP^+^, or FoxP3^-^LAP^+^ T cells. Proliferation of CTV labeled, αCD3/28 stimulated T_conv_ cells (33.46%) was significantly arrested when co-cultured with either FoxP3^+^LAP^+^ Tregs (16.82%) or FoxP3^-^LAP^+^ T cells (9.43%), but not when co-cultured with FoxP3^+^LAP^-^ Tregs (24.96%, **Figure 1D**).

Next, we confirmed earlier reports that FoxP3^+^ Tregs can be generated *in vitro* by culturing CD4^+^ T_conv_ cells with TCR stimulation and exogenous TGFβ supplementation (27), since 28.05±0.17% of T_conv_ cells stimulated with αCD3/28 microbeads in the presence of TGFβ had induced FoxP3 expression (**Figure 1E**). T_conv_ cells either mock stimulated or stimulated with αCD3/28 microbeads induced FoxP3 expression at low frequencies (2-2.54%). Co-culture of T_conv_ cells with FoxP3^+^LAP^-^ Tregs did not substantially induce FoxP3 expression (4.06±0.04% and 3.29±0.2%, αCD3/28 and αCD3 stimulation, respectively). In contrast, co-culture of T_conv_ cells with FoxP3^+^LAP^+^ Tregs and αCD3/28 significantly increased FoxP3 induction (4.7±0.08%). Co-culture with FoxP3^-^LAP^+^ T cells significantly increased FoxP3 induction when stimulated with αCD3 (4.60±0.2%), but not when stimulated with αCD3/28 microbeads (2.6±0.3%). Interestingly, we observed that upon co-culture with TCR stimulated T_conv_ cells, a significant fraction of FoxP3^-^LAP^+^ cells were induced to express FoxP3 (**Figure 1F**). Collectively, these findings indicate that FoxP3^+^LAP^+^ and FoxP3^-^LAP^+^ populations are induced by oral antigen gavage, produce IL-10, are highly suppressive, and can convert T_conv_ cells to a FoxP3 expressing phenotype. In this process, FoxP3^-^LAP^+^ cells can themselves convert to FoxP3^+^ cells.

### FoxP3^+^LAP^-^ and FoxP3^-^LAP^+^ cells exhibit distinct gene signatures

**Figure 2:**
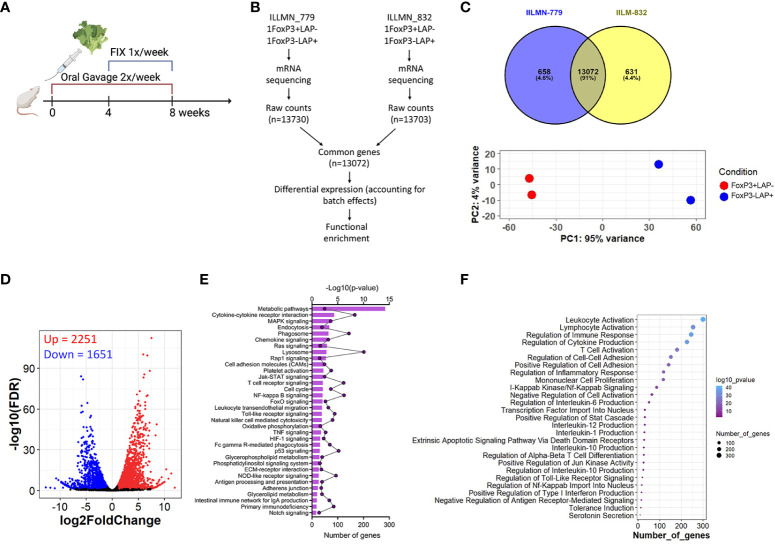
**(A)** Schema for mRNA sequencing experiment. HB mice (n=4/group) received FIX expressing lettuce administered 2X/week for 8 weeks by oral gavage. From weeks 4-8, animals additionally received weekly IV injections of rhFIX. **(B)** mRNA sequencing of FoxP3^+^LAP^-^ and FoxP3^-^LAP^+^ populations from 2 independent experiments. Raw mRNA counts were combined (accounting for batch effects) and processed for differential and functional gene enrichment studies. **(C)** Venn plots and principal component analysis indicating variance between the 2 cell populations and between the 2 independent experiments. **(D)** Differentially expressed genes (DEGs) between FoxP3^+^LAP^-^ and FoxP3^-^LAP^+^ populations. Significantly upregulated genes are shown in red and downregulated genes are shown in blue. **(E)** Functional Pathway enrichment showing the top 35 pathways identified for the DEGs based on number of genes. **(F)** Gene ontology (GO) analysis showing top 26 significantly enriched terms based on number of genes.

Although the phenotypic and molecular heterogeneity of FoxP3^+^ Tregs is well established (48–50), our knowledge of FoxP3^-^LAP^+^ T cells is more limited. We set out to test the molecular profile of FoxP3^-^LAP^+^ T cells by performing a discovery based, ad interim mRNA sequencing analysis of FoxP3^+^LAP^-^ and FoxP3^-^LAP^+^ cells magnetically enriched and sorted from spleens of FIX plant fed HB mice treated as shown in **Figure 2A**. Since FoxP3 is an intracellular protein, we used CD25 as a surrogate cell surface marker to sort for FoxP3 expressing cells as there is generally a good correlation between CD25 and FoxP3 expression in murine splenic Tregs (3) (**Supplementary Figure 2**). To obtain data from biological replicates and independent experiments, 2 sets of pooled (n=4), independent experiments were combined and processed to remove batch effects (**Figures 2A, B**). We identified 13072 (91%) common genes between experiment 1 and 2 (**Figure 2B**), validating the decision to combine the 2 experiments. Principle component analysis (PCA) confirmed low variance (4%) between the 2 independent experiments, and high variance (95%) between the FoxP3^+^LAP^-^ and FoxP3^-^LAP^+^ populations, indicating a clear segregation in gene signatures (**Figure 2C**). Following normalization by FPKM, analysis of the 13072 common genes identified 3902 differentially expressed genes (DEG), of which 2251 were upregulated and 1651 downregulated in FoxP3^-^LAP^+^ cells, as compared to FoxP3^+^LAP^-^ Tregs (**Figure 2D**). A complete list of DEGs can be found in **Supplemental Datasheet 1**.

Functional enrichment yielded 81 pathways. Among the top 30 pathways (% enrichment 7.46 – 0.68) were “metabolic pathways”, “cytokine-cytokine receptor interaction”, “MAPK signaling”, “endocytosis”, “phagosome”, “chemokine signaling pathway”, “lysosome”, “cell adhesion molecules”, “JAK-Stat signaling”, “TCR signaling”, “NFκB signaling”, and leukocyte transendothelial migration (**Figure 2E**). GO enrichment analysis returned 548 DE GO terms. The top 5 GO terms (% hits 7.8 – 6.4) were “leukocyte activation”, “positive regulation of immune system process”, “lymphocyte activation”, “cytokine production”, and “regulation of immune response” (**Figure 2F**). These functional terms correlate with our understanding of the role of FoxP3^-^LAP^+^ T cells corresponding to their origin in the GALT and MLN, activation, transendothelial migration and chemotaxis to the periphery, where they act to modulate ADA responses. Other interesting GO terms included IL-10 production, NFκB signaling, positive regulation of Jun kinase activity, regulation of alpha-beta T cell differentiation, negative regulation of TNF superfamily members, and regulation of transcription factor import into the nucleus. Interestingly, both Pathway analysis and GO terms included genes associated with phagosomes and endocytosis, antigen processing and presentation. A complete list of Pathway and GO terms can be found in **Supplemental Datasheet 1**.

**Figure 3:**
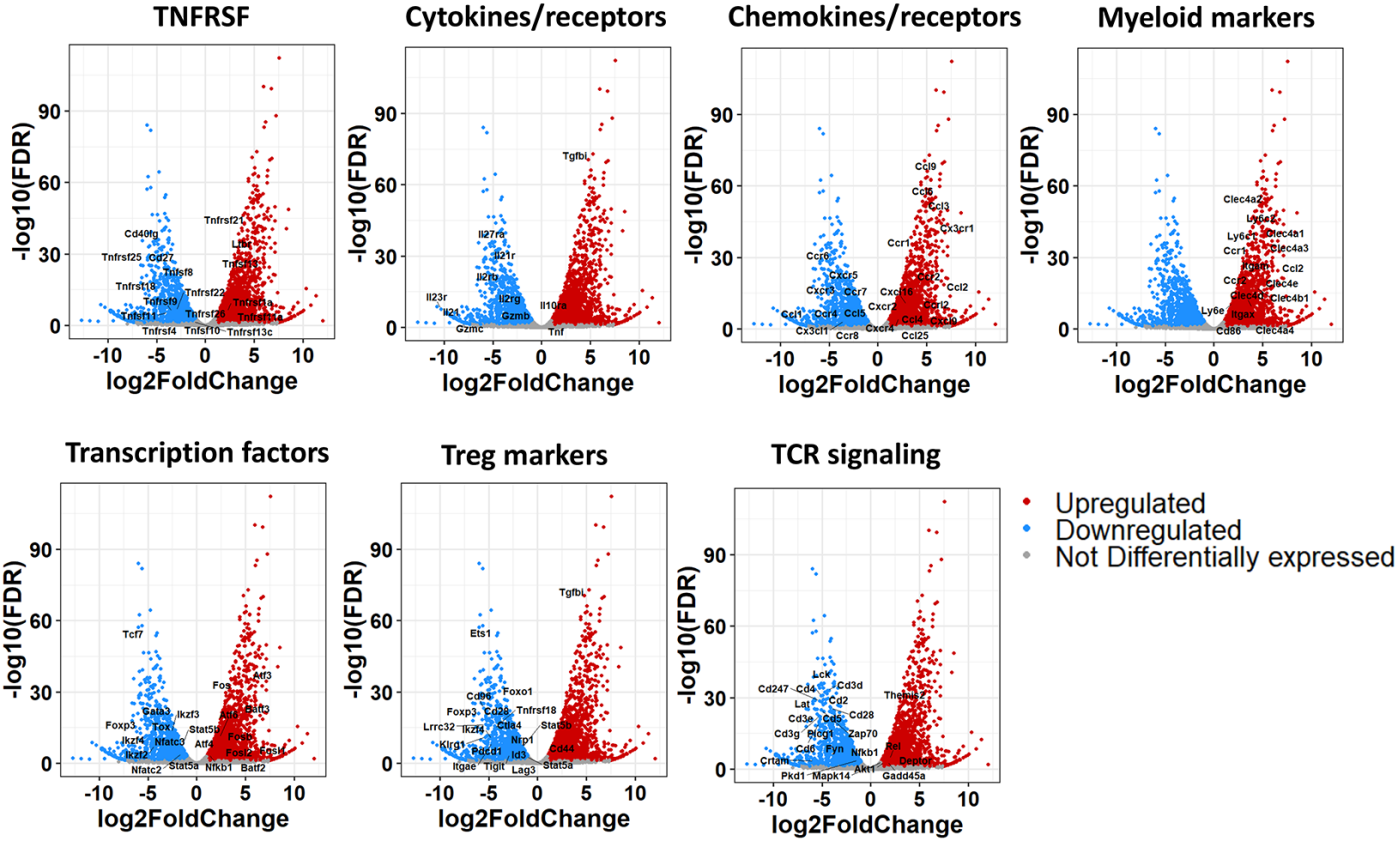
Volcano plots from mRNA seq analysis of FoxP3^+^LAP^-^ and FoxP3^-^LAP^+^ populations. DEGs from **Figure 2D** were further sub-grouped into TNF/TNFR superfamily, Cytokine/receptor, Chemokine/receptor, Myeloid, Transcription factor, Treg, and TCR signaling markers. Red and Blue indicate genes that are up or downregulated respectively in FoxP3^-^LAP^+^ compared to FoxP3^+^LAP^-^, and gray indicates genes that have no significant difference.

On analyzing DEGs, we confirmed that *Tgfbi* (LAP) was highly upregulated (log2 fold change = 5.27, adjusted p-value = 9.84E-74) and *Foxp3* downregulated (log2 fold change = -5.68, adjusted p-value = 4.04E-19) in the FoxP3^-^LAP^+^ population, which further validated the discrete identity of these 2 populations (**Supplemental Datasheet 1**). Based on the functional enrichment report and using an FDR of 0.05, we further curated DEGs based on observed patterns in TNF/TNFR superfamily (SF), cytokine/receptor, transcription factor, chemokine/receptor, FoxP3^+^ Treg associated markers, TCR signaling, and myeloid cell marker expression, represented as volcano plots (**Figure 3**). FoxP3^-^LAP^+^ T cells downregulated expression of TNF/TNFRSF members such as *Tnfrsf18* (GITR), *Tnfrfs25* (death receptor 3), *Tnfrsf9* (4-1BB), *Tnfrsf4* (OX40), *Tnfrsf26* (TNFRH3), *Tnfrsf22* (TRAIL), *Cd27* and *Cd40lg* (CD40L). Importantly, many of these downregulated TNF/TNFRSF members are costimulatory receptors that are constitutively expressed on FoxP3^+^ Tregs.

Cytokines and receptors associated with T cell activation or inflammation such as *Il2rb*, *Il2rg*, *Il21/Il21r*, *Il23r*, *Il27ra*, *Gzmb* and *Gzmc* were downregulated in FoxP3^-^LAP^+^ T cells, whereas *Tgfbi*, *Il10ra*, and *Tnf* were upregulated. We observed significantly increased expression of chemotactic molecules, notably *Ccl2* (MCP-1), *Ccl3* (MIP-1α), *Ccl4* (MIP), *Ccl9* (MIP-1γ), and receptors such as *Cx3cr1*, and *Ccr1/2* in FoxP3^-^LAP^+^ T cells. Interestingly, many of these chemokines and receptors are expressed in myeloid cells/macrophages and associated with cell migration. We therefore examined other myeloid cell markers and observed significantly upregulated expression of c-type lectins *Clec4a1/2/3/4*, *Clec4b1*, *Clec4d*, *Clec4e*, lymphocyte antigen (*Ly*) *6c1/2*, *Ly6e*, and integrins *Itgal* (CD11a), *Itgam* (CD11b), *Itgax* (CD11c), which are associated with monocytes/macrophages and/or dendritic cells (**Figure 3**).

FoxP3^-^LAP^+^ T cells also downregulated Treg associated transcription factors such as *Foxp3*, *Nfatc2/3*, *Stat5a/b*, Ikaros Zinc Finger (IKZF) family members such as *Ikzf2* (Helios), *Ikzf3* (Aiolos), *Ikzf4* (Eos), and T helper differentiating transcription factors such as *Gata3*, *Tcf7*, and *Tox*. Indeed, multiple transcription factors, co-inhibitory receptors, and other molecules associated with the development and function of FoxP3^+^ Tregs were downregulated in FoxP3^-^LAP^+^ T cells. Instead, we saw very high induction of members of the Activation Protein-1 (AP-1) transcription complex, such as *Fos*, *Fosb*, *Fosl1/2*, *Atf3/4*, *Batf3*, and *Nfkb1*. Finally, we observed downregulation of TCR-CD3 signaling complex members such as *Cd3d/g/e/z*, *Cd2*, *Cd4*, *Cd5*, *Cd6*, *Cd28*, *Zap70*, *Lck*, *Fyn*, *Lat*, and *Plcg1* (**Figure 3**). Overall, we observed that FoxP3^-^LAP^+^ cells had a unique transcriptomic profile that was distinct from that of FoxP3^+^LAP^-^ cells.

### FoxP3^+^LAP^-^, FoxP3^+^LAP^+^ and FoxP3^-^LAP^+^ cells differentially express phenotypic markers

**Figure 4:**
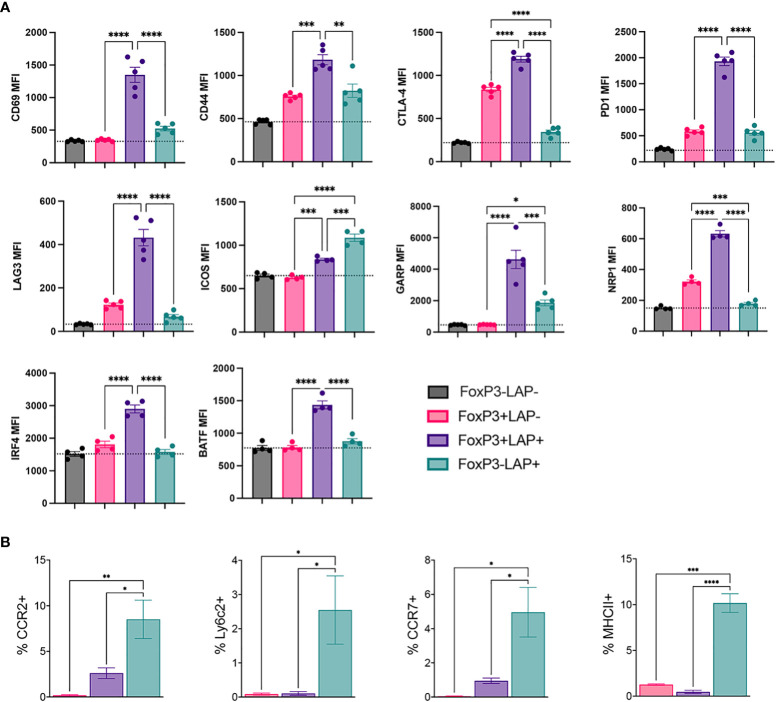
Flow cytometric analysis for phenotypic markers on regulatory T cell populations from hFVIII plant fed mice. HA mice HA mice (n=5/group) orally received FVIII HC/C2 expressing plant material and weekly rhFVIII injections as detailed in **Figure 1A**. **(A)** Median fluorescence intensity (MFI) of CD69, CD44, CTLA-4, PD1, LAG3, ICOS, GARP, NRP1, IRF4, and BATF in FoxP3^-^LAP^-^ (T_conv_), FoxP3^+^LAP^-^, FoxP3^+^LAP^+^ and FoxP3^-^LAP^+^ populations. **(B)** MFI of CCR2, Ly6C2, CCR7 and MHCII expression in FoxP3^+^LAP^-^, FoxP3^+^LAP^+^ and FoxP3^-^LAP^+^ populations. Statistical significance was calculated by one way ANOVA with Tukey’s *post hoc* test for 4A and 4B. *p ≤ 0.05 **p ≤ 0.01; ***p ≤ 0.001; ****p ≤ 0.0001.

We performed flow cytometry analysis to validate downregulation of the Treg phenotype in the FoxP3^-^LAP^+^ population. We compared T_conv_ (FoxP3^-^LAP^-^), FoxP3^+^LAP^-^, FoxP3^+^LAP^+^, FoxP3^-^LAP^+^ cells (n=4/group). The FoxP3^+^LAP^+^ population highly upregulated activation and co-inhibitory molecules such as CD69, CD44, CTLA-4, PD1, LAG3, ICOS, GARP, and NRP1, consistent with its role as an activated cell subset with superior suppressive function (12, 33) (**Figure 4A**). This population also upregulated the transcription factors IRF4 and BATF, which initiates differentiation towards an effector Treg phenotype (51). FoxP3^+^LAP^-^ cells upregulated signature Treg markers, such as CTLA-4 and NRP1, although CD69 and CD44 expression was not upregulated, indicating that the bulk of these Tregs were not specific for the FVIII antigen. In line with the mRNA sequencing analysis in **Figure 3**, FoxP3^-^LAP^+^ T cells had lower expression of co-inhibitory receptors CTLA-4, PD1, LAG3, and NRP1 (52, 53). Instead, FoxP3^-^LAP^+^ T cells upregulated the costimulatory receptor ICOS, and the TGFβ binding protein GARP (*lrrc32*), which anchors LAP to the cell surface (**Figure 4A**).

Interestingly, the FoxP3^-^LAP^+^ population upregulated CCR2, Ly6C2, CCR7 and MHCII, which are more traditionally associated with monocytes/macrophages, and were virtually absent on the FoxP3^+^LAP^-^, and FoxP3^+^LAP^+^ populations (**Figure 4B**). These data validated the mRNA sequencing analysis, further indicating that FoxP3^-^LAP^+^ T cells have a separate phenotype from that of FoxP3 expressing cells.

### Single cell RNA sequencing reveals highly distinguishing characteristics between but also heterogenicity within Treg populations, with LAP^+^FoxP3^+^ Treg representing an activated form of FoxP3^+^ Treg

**Figure 5:**
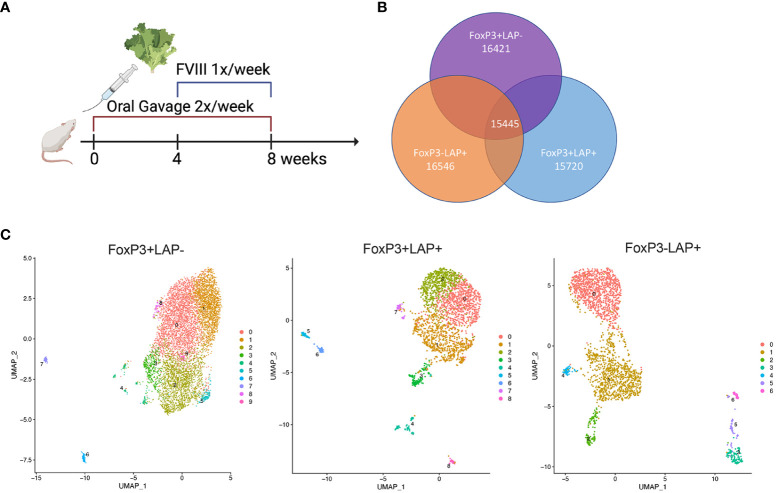
scRNA seq analysis of regulatory T cell populations in HA mice. **(A)** Timeline. HA mice (n=4) orally received hFVIII HC/C2 expressing lyophilized lettuce reconstituted in PBS, administered 2X/week for 8 weeks. From weeks 4-8, animals additionally received weekly IV injections of rhFVIII. **(B)** Venn diagram representing the number of overlapping and nonoverlapping genes between FoxP3^+^LAP^-^, FoxP3^+^LAP^+^, and FoxP3^-^LAP^+^ populations from **(A)** recovered at 8 weeks. **(C)** UMAP visualization of transcriptomes from FoxP3^+^LAP^-^, FoxP3^+^LAP^+^, and FoxP3^-^LAP^+^ populations, showing 10, 9 and 7 clusters respectively.

**Figure 6:**
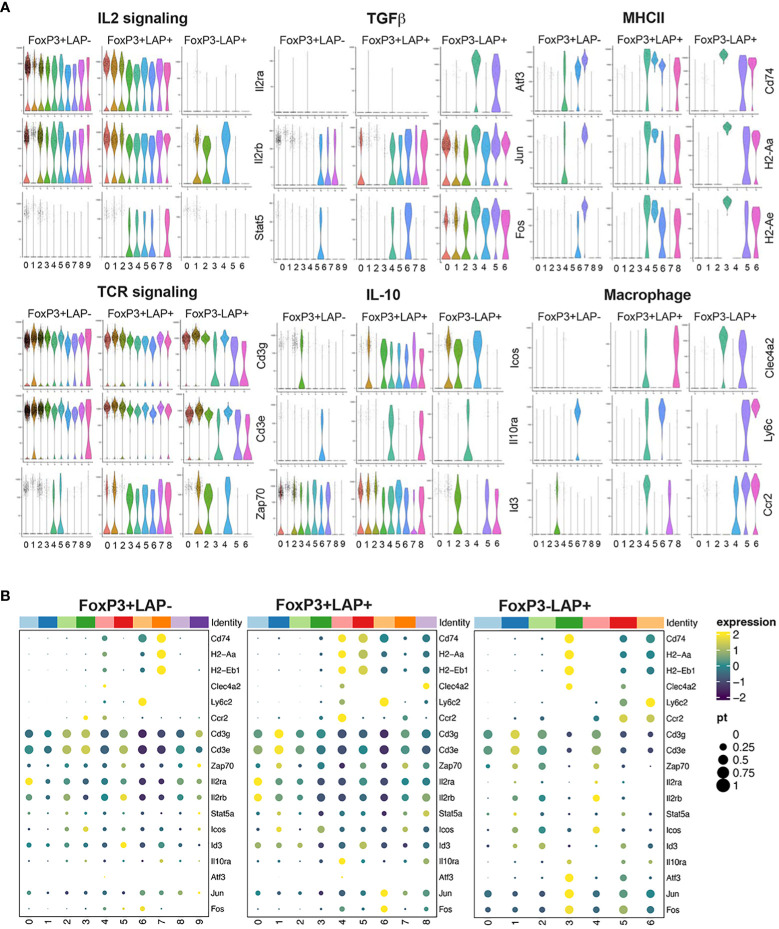
Individual UMAP analysis of FoxP3^+^LAP^-^, FoxP3^+^LAP^+^ and FoxP3^-^LAP^+^ populations. Representative genes from selected pathways for UMAP derived clusters of FoxP3^+^LAP^-^, FoxP3^+^LAP^+^, and FoxP3^-^LAP^+^ populations presented as **(A)** Violin plots and **(B)** Cluster dot plots.

To better understand the contribution of CD4^+^ T cell subsets toward oral tolerance to FVIII, we performed an in-depth single cell transcriptomic study in HA mice. Animals received FVIII expressing plant followed by IV rhFVIII injections (**Figure 5A**). At the end of the treatment, splenocytes were isolated and single cell RNA (scRNA) library prep and sequencing was performed on 2 cell sorted populations: CD4^+^FoxP3^+^ and CD4^+^FoxP3^-^LAP^+^ using CD25 as a surrogate marker for FoxP3 expression. Pre- and post sort purity of the 2 populations is indicated in **Supplementary Figure 2**. Data is available in GEO under accession GSE242919. Reads were further filtered for FoxP3 or LAP expression to remove contaminating cells using differential expression analysis by LTMG software (54, 55), which is a robust algorithm that can handle batch effect in comparing expression across different samples. Next, we further separated the CD4^+^FoxP3^+^ subset into 2 populations based on LAP expression: FoxP3^+^LAP^-^ and FoxP3^+^LAP^+^ (**Supplementary Figure 2**). We recovered 16421, 16546, and 15720 genes from FoxP3^+^LAP^-^, FoxP3^+^LAP^+^, and FoxP3^-^LAP^+^ populations respectively, with 15445 common genes between the 3 populations (**Figure 5B**). We performed individual analysis of the 3 populations by unsupervised clustering and dimension reduction in Seurat, and visualization using a uniform manifold approximation and projection (UMAP) method (56) (**Figure 5C**). FoxP3^-^LAP^+^, FoxP3^+^LAP^-^, and FoxP3^+^LAP^+^ cells segregated into 10, 9, and 7 clusters respectively. Within the FoxP3^-^LAP^+^ population, clusters 3, 5, and 6 showed distinct gene expression profiles consistent with the mRNA seq and flow cytometry data from **Figures 3**, **4** (**Supplemental Datasheet 2**). Based on the exploratory data generated from bulk mRNA-seq analysis (**Figure 3**), we concentrated on genes from 6 major pathways: IL2 signaling, TCR signaling, TGFβ, IL-10, MHCII, and myeloid cell markers [harmonizome curated database (57)]. Detailed scatter plots of genes for individual pathways are provided in **Supplementary Figure 3**. Violin plots and scatter dot plots of representative genes from the FoxP3^-^LAP^+^ population showed downregulation of IL2 and TCR-CD3 signaling genes, such as *Il2ra*, *Il2rb*, *Stat5*, *Cd3d/e* and *Zap70*, particularly in clusters 3, 5, and 6 (**Figures 6A, B**). These same clusters showed 10- or 100- fold higher upregulation of the TGFβ associated AP-1 transcription complex, in comparison to the FoxP3^+^LAP^-^ and FoxP3^+^LAP^+^ populations, respectively (**Figures 6A, B**, **Supplementary Figure 3** and **Supplemental Datasheet 2**). Additional genes in the TGFβ pathway were also upregulated in these clusters, such as *Junb*, *Jund*, *Tgbr1/2* (**Supplementary Figure 3**). Clusters 3, 5, and 6 in the FoxP3^-^LAP^+^ population also upregulated MHCII associated genes such as *H2-A/D/E*, *Cd74* and *Ciita*, and myeloid cell associated members such as *Clec4a2*, *Ly6c*, and *Ccr2* (**Figures 6A, B**, **Supplementary Figure 3** and **Supplemental Datasheet 2**). Clusters 3, 5, and 6 also did not express T cell differentiation transcription factors such as *Irf4*, *Gata3*, *Tbx21*, *Tcf7*, *Prdm1* (Blimp1), or *Bcl6*.

FoxP3^+^LAP^+^ population represented an activated subset of FoxP3^+^ Tregs, upregulating markers that correlate with Treg activation and/or suppressive function such as *Lgals1*, *Ctla4*, *Cd44*, *Irf4*, *Batf*, *Tnfrsf1b* (TNFR2), *Tnfrsf18*, *Tnfrsf4*, *Tnfrsf9*, *Ikzf2*, *Hif1a*, *Maf*, *Gata3*, *Nfkb1*, *Icos*, *Cd3g/d/e* (**Supplemental Datasheet 2**). Multiple clusters of FoxP3^+^LAP^+^ cells strongly expressed *Tgfb1*, upregulated IL2 receptor and TCR signaling pathways, and engaged TGFβ and IL-10 pathways (**Figures 6A, B**; **Supplementary Figure 3**). Interestingly, in the FoxP3^+^LAP^+^ population, common clusters were responsible for upregulation of IL2Rα and TCR signaling, as well as TGFβ and IL-10 pathways. This indicates that immune modulatory cytokine production can be induced via both FoxP3, TCR and IL2 receptor dependent and independent mechanisms in different subsets of CD4^+^ T cells (**Figures 6A, B**). In contrast, the FoxP3^+^LAP^-^ population had a broadly resting phenotype (lower *Stat5* and *Zap70* expression, lower expression of TGFβ or IL-10 pathway intermediates), again indicating that the bulk of FoxP3^+^LAP^-^ cells were not FVIII antigen specific (**Figures 6A, B**, **Supplementary Figure 3**, **Datasheet 2**).

**Figure 7:**
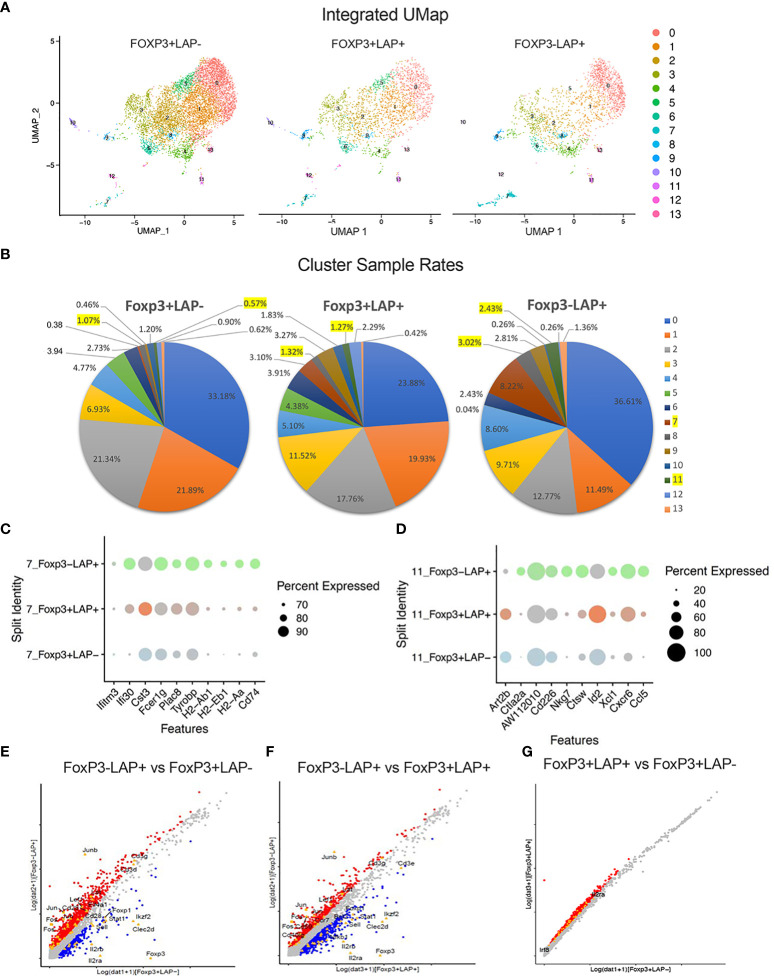
Integrated UMAP analysis of FoxP3^+^LAP^-^, FoxP3^+^LAP^+^ and FoxP3^-^LAP^+^ populations. **(A)** UMAP plots showing segregation of FoxP3^+^LAP^-^, FoxP3^+^LAP^+^ and FoxP3^-^LAP^+^ populations into 14 clusters. **(B)** Cluster sample rates indicating relative frequencies of the 14 UMAP clusters in FoxP3^+^LAP^-^, FoxP3^+^LAP^+^ and FoxP3^-^LAP^+^ populations. Clusters 7 and 11 are highlighted. **(C)** Dot plots showing comparative representation of the top 10 overexpressed genes from Cluster 7 in the 3 populations. Size of the dot corresponds to the percentage of cells expressing the gene in each cluster. **(D)** Dot plot showing comparative representation of the top 10 overexpressed genes from Cluster 11 in the 3 populations. Size of the dot corresponds to the percentage of cells expressing the gene in each cluster. **(E–G)** DEG analysis. Scatterplots depicting up- and downregulated genes between **(E)** FoxP3^-^LAP^+^ and FoxP3^+^LAP^-^, **(F)** FoxP3^-^LAP^+^ and FoxP3^+^LAP^+^, and **(G)** FoxP3^+^LAP^+^ and FoxP3^+^LAP^-^ populations. Selected immune associated genes are indicated. Red and Blue dots indicate genes that are up or downregulated respectively and gray indicates genes that have no significant difference.

To uncover additional differences between these 3 populations, we performed an integrated UMAP analysis of FoxP3^-^LAP^+^, FoxP3^+^LAP^-^, and FoxP3^+^LAP^+^ cells, which generated 14 clusters (**Figure 7A**). On analyzing the relative frequencies of each population, we observed significant enrichment in cluster 7 (3.02%) and 11 (2.43%) in the FoxP3^-^LAP^+^ population, which was moderately expressed in the FoxP3^+^LAP^+^ population (1.32% and 1.27%), and negligibly expressed in the FoxP3^+^LAP^-^ population (1.07% and 0.57%) (**Figure 7B**). Cluster 7 predominated with MHCII and myeloid gene markers, such as *H2-A/D/E*, *Cd74*, *Ifitm*, *Clec4/9/12*, *Ly6c2*, *Ccr2*, *Cd68*, *Itgax*, whereas Cluster 11 had increased expression of T cell activation and infiltration genes such as *Ccl5*, *Cxcr6*, *Xcl1*, *Ctla2a*, *Cd226*, *Cd40Ig*, and *Icos*. The top 10 genes for Clusters 7 and 11 are shown in **Figures 7C, D** respectively and completely listed in **Supplemental Datasheet 3**.

Next, we performed direct comparisons between populations using differential expression and analysis. We identified 1578-upregulated and 273-downregulated genes in the FoxP3^-^LAP^+^ vs FoxP3^+^LAP^-^ population (**Figure 7E**). We also identified 768-upregulated and 739-downregulated genes in the FoxP3^-^LAP^+^ vs FoxP3^+^LAP^+^ (**Figure 7F**), 1647-upregulated and 0- downregulated genes in the FoxP3^+^LAP^+^ vs FoxP3^+^LAP^-^ population (**Figure 7G**). As observed with the mRNA seq DEG analysis, FoxP3^-^LAP^+^ highly upregulated the AP1-Jun-Fos pathway as compared to either FoxP3^+^LAP^-^ or FoxP3^+^LAP^+^ populations. Among the top 50 upregulated genes were *Atf3*, *Tgfb1*, *Junb*, *Fos*, *Jun*, *Dusp1*, *Cd69*, *Klf6*, *Nfkbia*, *Nfkb1*, and *Ccr7*. Among the top 50 FoxP3^-^LAP^+^ downregulated genes were Treg signature markers such as *Foxp3*, *Il2ra*, *Clec2d*, *Il2rb*, *Ikzf2*, *Izumo1r*, *Ikzf4*, *Prkca*, *Socs1*, *Il27ra*, *Sell*, and *Stat1*. We also confirmed downregulation of TNF/TNFR superfamily members such as *Tnfrsf18*, *Tnfrsf4*, *Tnfrsf9*, *Tnfrsf1b*, similar to what was observed in **Figure 3**.

Relative to the FoxP3^+^LAP^-^ population, 1504 genes were differentially upregulated in the FoxP3^+^LAP^+^ population (using 0.25 log2 fold change as a cut off). Analysis of differentially expressed genes confirmed an activated Treg phenotype, and upregulation of TGFβ associated genes in the FoxP3^+^LAP^+^ population (e.g. *Lgals1*, *Il2ra*, *Il2rb*, *Il2rg*, *Cd28*, *Icos*, *Cd44*, *41bb*, *Fr4* or *Izumo1r*, *Irf4*, *Nfkb*, *Maf*, *Nfat*, *Smad4*, *Junb*, *Jund*, *Batf*, *Atf4*, among others). Interestingly, FoxP3^+^LAP^+^ did not downregulate any FoxP3^+^ associated genes, confirming that this cell type retained a Treg phenotype, while upregulating activation markers (**Figure 7G**). An extended list of DEGs is provided in **Supplemental Datasheet 4**.

## Discussion

Understanding cell-type-specific signatures and regulation at the single-cell level enables decoding of the molecular mechanisms contributing to the function of T cell subsets. In this work, we integrated mRNA sequencing, scRNA sequencing, flow cytometry and functional assays to delineate subsets of orally induced Treg involved in systemic suppression of ADAs. We sought to characterize orally induced Treg subsets in the spleen, which we recently identified as the site of initiation of ADA responses to clotting factor replacement therapy (17).

### LAP^+^FoxP3^-^ and FoxP3^+^ Treg have distinct gene expression patterns and characteristics

Our findings indicate that FoxP3^-^LAP^+^CD4^+^ T cells are a highly suppressive and phenotypically distinct cell subset with a unique gene signature, characterized by low expression of genes involved in TCR signaling or costimulation (except for ICOS), upregulated expression of MHC II genes, and migratory receptor expression. These FoxP3^-^LAP^+^ cells were most effective in arresting proliferation of αCD3/28 stimulated T_conv_ cells, had the capacity to induce TCR activated T_conv_ cells to express Foxp3, may themselves convert to FoxP3^+^ cells, and produced IL-10 upon stimulation. In contrast, FoxP3^+^LAP^+^ dual positive cells represent an activated form of traditional FoxP3^+^ Treg (rather than a distinct subset) that acquired those gene expression characteristics of FoxP3^-^LAP^+^ cells that relate to TGF-β and ICOS expression. Both these suppressive cell types expressed surface LAP, supporting prior claims in the literature that TGFβ complexed to LAP is sufficient to induce FoxP3 expression in a contact dependent manner (12, 13, 33, 58–61).

FoxP3 interacts with TCR‐induced transcription factors, which are activated through signaling cascades initiated by TCR and CD28 costimulatory signals. Consistent with this, FoxP3^+^LAP^+^ cells expressed high levels of *Lgals1*, *Pdcd1*, *Ctla4*, *Cd69*, *Cd44*, and *Nrp1*, molecules whose expression can be induced by and/or maintained by persistent TCR engagement (62, 63), as well as the transcription factor *Irf4* and its partner *Batf*, contributing to the generation of an effector Treg population with superior suppressive capacity (51, 64). Consistent with TCR/CD28 activation, FoxP3^+^LAP^+^ cells also highly upregulated TNFRSF members such as *Tnfrsf18*, *Tnfrsf4*, *Tnfrsf9*, and *Tnfrsf1b*, which have been shown to increase Treg activation and function via *Nfkb* activation (65). TCR signaling, co-inhibitory receptors and TNFRSF members were mostly downregulated in FoxP3^-^LAP^+^ cells, in concordance with studies supporting the hypoproliferative nature of these cells (24, 26).

### Role of IL-10 expression

A common mediator of suppression by FoxP3^+^ Tregs is IL-10 or IL-35 production (66). We confirmed at the protein level that both *in vitro* stimulated LAP expressing FoxP3^+^ and FoxP3^-^ cells produced IL-10, with FoxP3^-^LAP^+^ cells constituting the largest population of IL-10 producers. We and others have previously shown that suppression by FoxP3^-^LAP^+^ T cells is driven by a complex process that depends on IL-10 production (13, 67, 68). Consistent with this, we observed high abundance of *Icos* mRNA and surface ICOS expression in FoxP3^+^LAP^+^ and FoxP3^-^LAP^+^ cells. ICOS can induce IL-10 production in both T_conv_ and Tregs, contributing to the induction of anergy and development of suppressive T cells (69). Other components involved in IL-10 production such as *Jun*/*AP-1 (* 70), *Cebpb*, and *IL10ra/b* were also upregulated in FoxP3^+^LAP^+^ and FoxP3^-^LAP^+^ cells (71, 72). However, we did not detect transcripts for *Il10* by mRNA sequencing or by single cell RNA sequencing. This may be attributed to the short half-life of cytokine transcripts (73), or the kinetics of IL-10 expression as analyses were performed 4 weeks after the last antigen administration. CD25^-^LAP^+^ cells also appear to be distinct from another suppressive T cell subset with high IL-10 production capacity: CD49b^+^LAG3^+^FoxP3^-^ Tr1 cells.

### Role of TGFβ and LAP

LAP^+^ cells hold TGFβ in an inactive state, either bound to the extra-cellular matrix (ECM) by LTBP or tethered to the cell membrane by *Lrrc32* (GARP) (74). While both FoxP3^+^ and FoxP3^-^ cells can express LAP, its expression appears to be induced by different mechanisms. In FoxP3^+^ cells, IL2Rα is upregulated upon TCR activation (75). Binding of AP-1 members such as *JunB* to the IL2Rα promoter can directly induce LAP or TGFβ expression, as well as the expression of other Treg related genes, such as *Ctla4*, *Socs2*, *Lgals1*, *Ccr4*, and *Gpr83* (76, 77). In this case, AP-1 members such as *JunB* play an important role in Treg differentiation via upregulation of IRF, BATF and IL2Rα expression, as observed in the FoxP3^+^LAP^+^ population (76, 78, 79). Conversely, TGFβ can also induce FoxP3 expression in T_conv_ cells by recruitment of activated Smad3 to the FoxP3 promoter via an enhanceosome (80). This process requires IL-2, which allows STAT5 to bind to the FoxP3 promoter to induce FoxP3 transcription (81–83). FoxP3^-^LAP^+^ cells, on the other hand, do not produce IL-2 and downregulate IL2Rα/β/γ expression.

The AP-1 complex is comprised of heterodimers of ATF, Jun, Fos, and Maf sub-families (84). Binding of the AP-1 complex to the TGFβ promoter can transactivate TGFβ expression, and TGFβ has been shown to stimulate the expression of AP-1 genes in various studies (77, 85). AP-1 can also bind to the *IL10* locus through the cooperative function of Fos/Jun family proteins, and Atf3 and Fosl2 have been identified as positive regulators of IL10 production (71, 86). *Atf3* is an adaptive response gene, whose upregulation is induced by various stress signals or by TGFβ and can upregulate the expression of the *Tgfb* gene itself, forming a positive-feedback loop for TGFβ signaling (87). Interestingly, we see recruitment of the AP-1 transcriptional complex at even higher transcriptional levels in the FoxP3 and IL2Rα independent induction of LAP, especially *Atf3*, *Jun*, *Junb*, *Jund*, *Fos*, and *Fosl* transcripts. *Atf3* in particular was highly upregulated in both the mRNA and scRNA sequencing datasets. *Atf3* has been shown to protect against colitis by regulating T follicular helper (Tfh) cells in the gut, leading to greatly diminished germinal center cells in Peyer’s patches (88). Since inhibitor development in hemophilia depends on Tfh cells and a productive germinal center response, this link between *Atf3* and Tfh cell survival should be further explored. It should be remarked that both FoxP3^-^LAP^+^ and FoxP3^+^LAP^+^ cells upregulate the AP1-Jun/Fos pathway, indicating that LAP/TGFβ production via AP1-Jun/Fos signaling is requisite for oral tolerance induction in both FoxP3^+^ and FoxP3^-^ T cells.

### Potential implications for co-stimulation and migration

An important mechanism of suppression by both FoxP3^+^ and Tr1 cells is the constitutive expression and upregulation of co-inhibitory receptors such as CTLA-4, PD1, or LAG3, which can compete with costimulatory molecules such as CD28 for ligation to CD80/86 on APC (89). These co-inhibitory receptors were found to be downregulated on FoxP3^-^LAP^+^ T cells. Considering that co-inhibitory receptor binding to antigen presenting cells is a major mechanism of contact dependent suppression by FoxP3^+^ Tregs, it would be important to understand how FoxP3^-^LAP^+^ T cells exert a contact dependent suppressive effect. A recent study showed that LAP expressing γδ T cells can express MHCII, CD40 and CD86, and can function as antigen presenting cells that induce CD4^+^Foxp3^+^ regulatory T cells (31). We observe a similar upregulation of MHCII associated markers in our study, as well as receptors such as CCR2, Ly6C, CD11c etc. While these markers are traditionally associated with myeloid cells, they may also reflect T cell activation and/or migration. As documented in other experimental models, we propose that gut induced cells migrate into secondary lymphoid organs in response to systemically delivered antigen (90, 91). Consistant with this notion, we also observe upregulation of other receptors that promote trafficking into secondary lymphoid organs such as *Cd62l* (Sell), and *Ccr7* in the FoxP3^+^LAP^+^ and FoxP3^-^LAP^+^ populations (92). However, migration of orally induced cells between GALT, MLNs and periphery is not as well studied for FoxP3^-^LAP^+^ Treg as it is for FoxP3^+^ Treg. Therefore, our hypothesis that the pattern of cell surface receptor expression that we observed in the Treg subsets reflects migration and homing to lymphoid organs in response to systemic antigen challenge awaits further experimental confirmation.

## Conclusions

**Figure 8:**
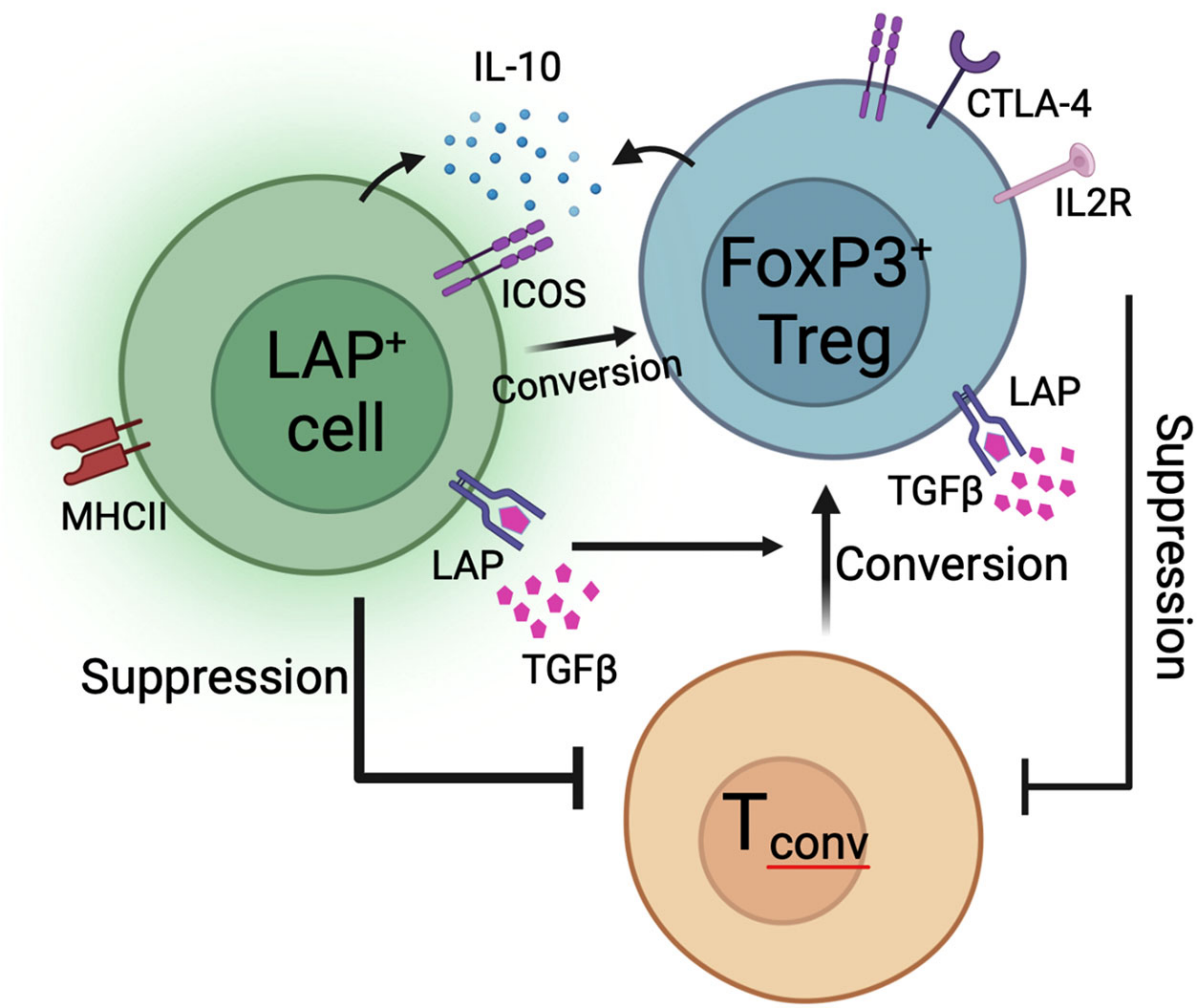
Proposed model of oral tolerance to ADA formation. FoxP3^-^LAP^+^ cells are characterized by expression of LAP, ICOS, MHCII, and produce IL-10 and TGFβ. FoxP3^+^LAP^+^ Treg also express LAP, ICOS, and additionally upregulate co-inhibitory receptors like CTLA-4 and IL2Rα (CD25). Both FoxP3^-^LAP^+^ and FoxP3^+^LAP^+^ cells can suppress the activation and proliferation of T_conv_ cells. Moreover, both cell types can catalyze the conversion of T_conv_ cells into FoxP3^+^ Tregs via contact/non-contact dependent mechanisms. FoxP3^-^LAP^+^ cells can themselves convert into FoxP3^+^ Tregs.

In conclusion, we prose a model in which orally induced Treg subsets migrate from the gut into secondary lymphoid organs to suppress ADA responses to systemically delivered antigen (**Figure 8**). Suppression is dependent on the expression of TGFβ that is either soluble or bound to the cell surface to exert contact dependent suppression. Contact dependent suppressive mechanisms may be enhanced by MHCII expression, although this mechanism remains to be determined. Production of IL-10 by FoxP3^-^LAP^+^ and FoxP3^+^LAP^+^ cells enhances TGFβ mediated conversion of T_conv_ cells into FoxP3^+^ antigen specific Tregs. In this process, FoxP3^-^LAP^+^ cells can themselves convert into FoxP3^+^ Tregs. It is difficult to completely delineate functions between FoxP3^-^LAP^+^ and FoxP3^+^LAP^+^ populations, as the induction and mechanisms of suppression by these two populations appear to be interconnected and thus inseparable. Whereas we have previously shown that adoptive transfer of a very small population of FoxP3^-^LAP^+^ cells are potently suppressive (15), this effect is likely not independent, but ultimately comprises a concerted effort by FoxP3^-^ and FoxP3^+^ cells.

## Data availability statement

The data presented in the study are deposited in the GEO repository, accession number GSE242919.

## Ethics statement

The animal study was approved by Indiana University School of Medicine IACUC. The study was conducted in accordance with the local legislation and institutional requirements.

## Author contributions

RH: Conceptualization, Funding acquisition, Investigation, Project administration, Supervision, Writing – original draft, Writing – review & editing. MB: Conceptualization, Data curation, Formal Analysis, Investigation, Methodology, Supervision, Writing – original draft, Writing – review & editing. KS: Data curation, Formal Analysis, Methodology, Software, Writing – review & editing. TB: Data curation, Investigation, Methodology, Writing – review & editing. PK: Data curation, Formal Analysis, Methodology, Software, Writing – review & editing. JR: Investigation, Writing – review & editing. MM-M: Investigation, Writing – review & editing. FS: Investigation, Writing – review & editing. SK: Investigation, Writing – review & editing. HG: Data curation, Methodology, Software, Writing – review & editing. XX: Data curation, Methodology, Software, Writing – review & editing. CT: Conceptualization, Funding acquisition, Supervision, Writing – review & editing. HD: Conceptualization, Funding acquisition, Supervision, Writing – review & editing. SC: Methodology, Supervision, Writing – review & editing.
