# Supplementary material for: Distinct functions and transcriptional signatures in orally induced regulatory T cell populations
Source: Front Immunol. 2023 Oct 26;14:1278184. doi: 10.3389/fimmu.2023.1278184 (PMC10637621; doi:10.3389/fimmu.2023.1278184)
Supplement: Supplementary file 5 [file Image_1.pdf]

Supplementary Fig.1

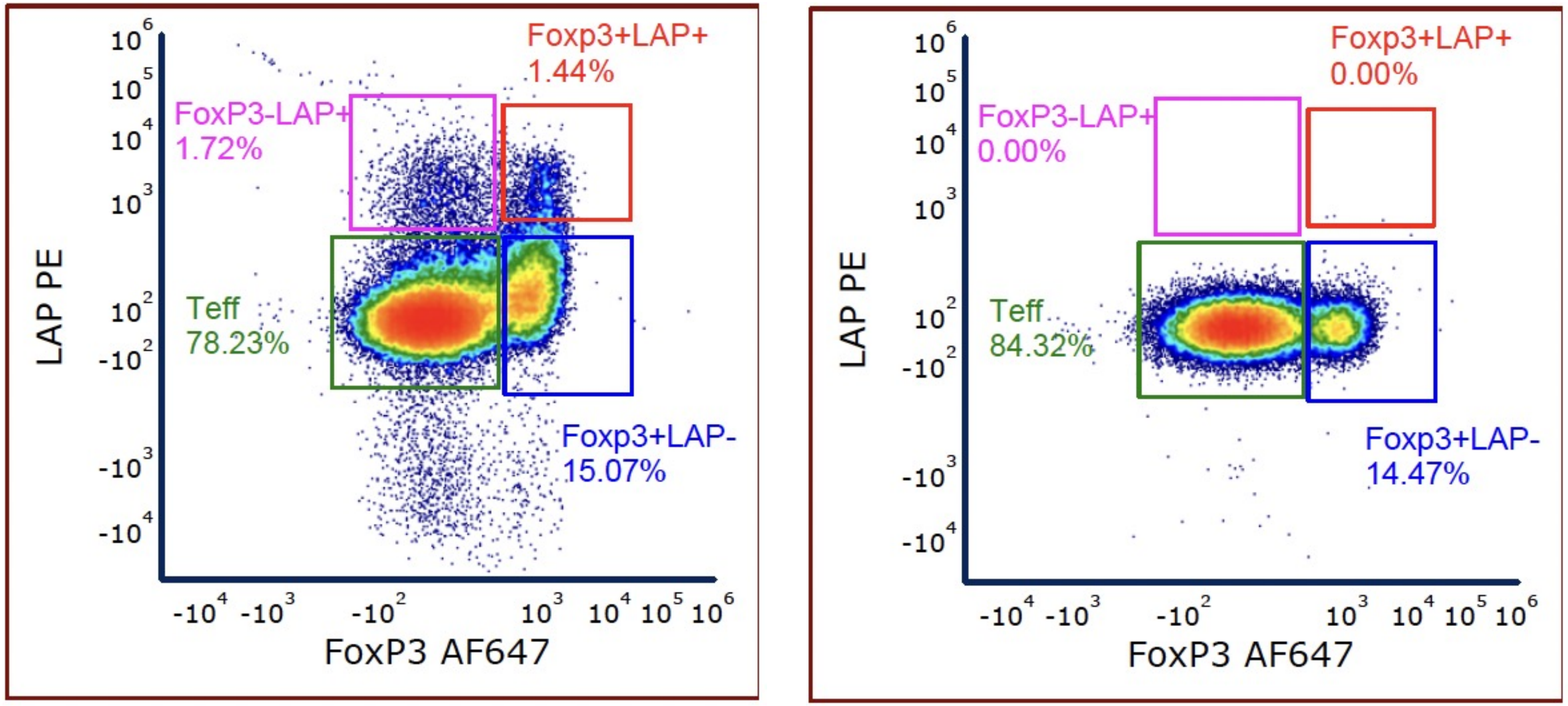

Representative density plots indicating gating scheme for identification of FoxP3^{+}LAP^{-}, FoxP3^{+}LAP^{+}, and FoxP3^{-}LAP^{+} populations. Isotype control for LAP is used to indicate antibody specificity.

Supplementary Fig.2

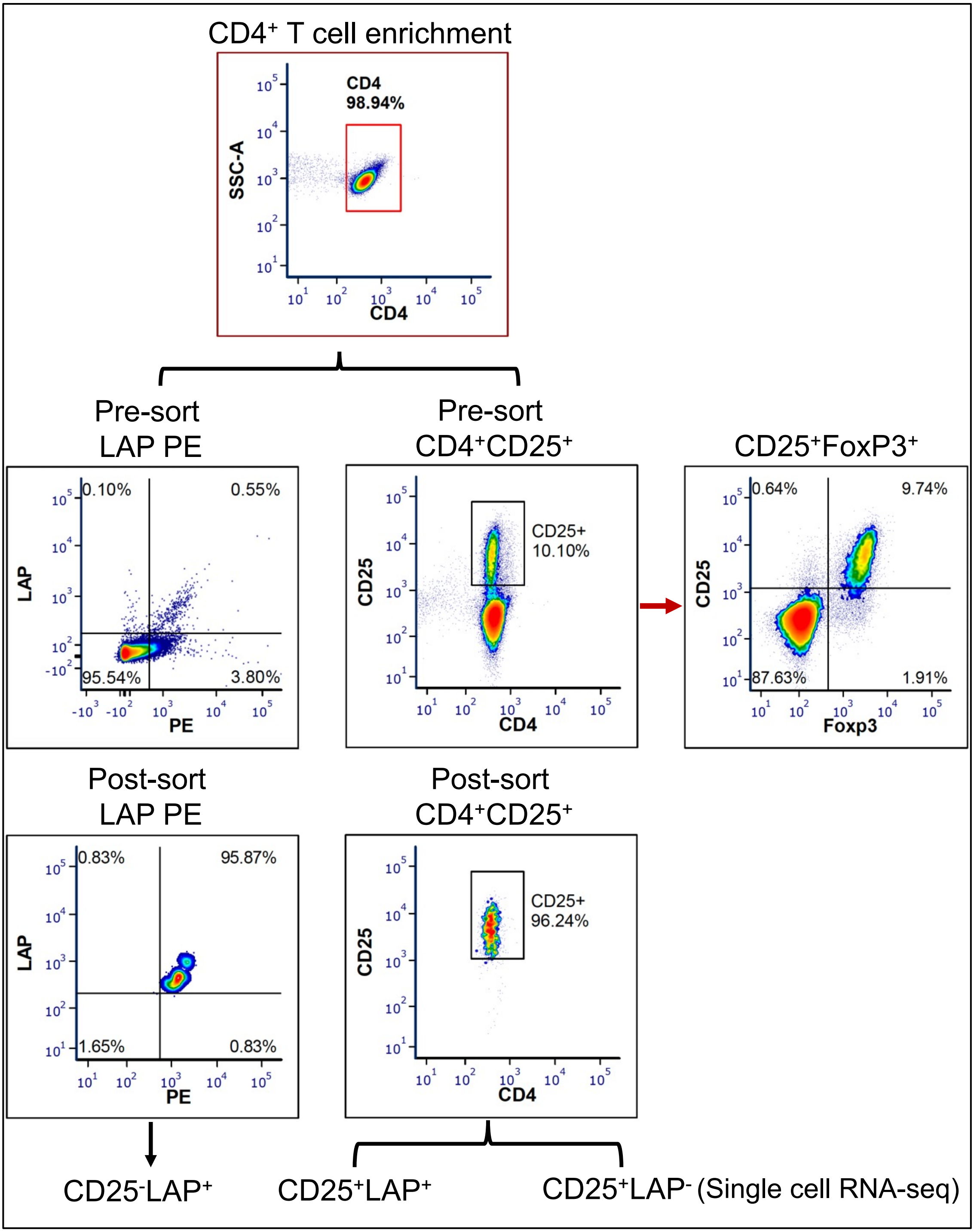

Pre- and post- sort purity analysis. Magnetically enriched CD4⁺ T cells are either incubated with LAP-PE conjugated antibody or further enriched for CD25⁺ Tregs. Intracellular staining confirms correlation between CD25 and FoxP3 expression. Only a negligible population of CD25⁺FoxP3⁻ cell are observed. Post- sort analysis shows ~96% pure CD25⁻LAP⁺ cells, and ~96% CD25⁺ cells. During single cell RNA analysis, CD25⁺ cells are further filtered into CD25⁺LAP⁺ (FoxP3⁺LAP⁺) and CD25⁺LAP⁻ (FoxP3⁺LAP⁻) populations.

Supplementary Fig.3

Scatter dot plots for FoxP3$^+$LAP$^-$, FoxP3$^+$LAP$^+$, and FoxP3$^-$LAP$^+$ clusters indicating enrichment of genes associated with IL-2, TGFβ, TCR, MHCII, myeloid, and IL-10 pathways.

IL-2 Clustered dot plot

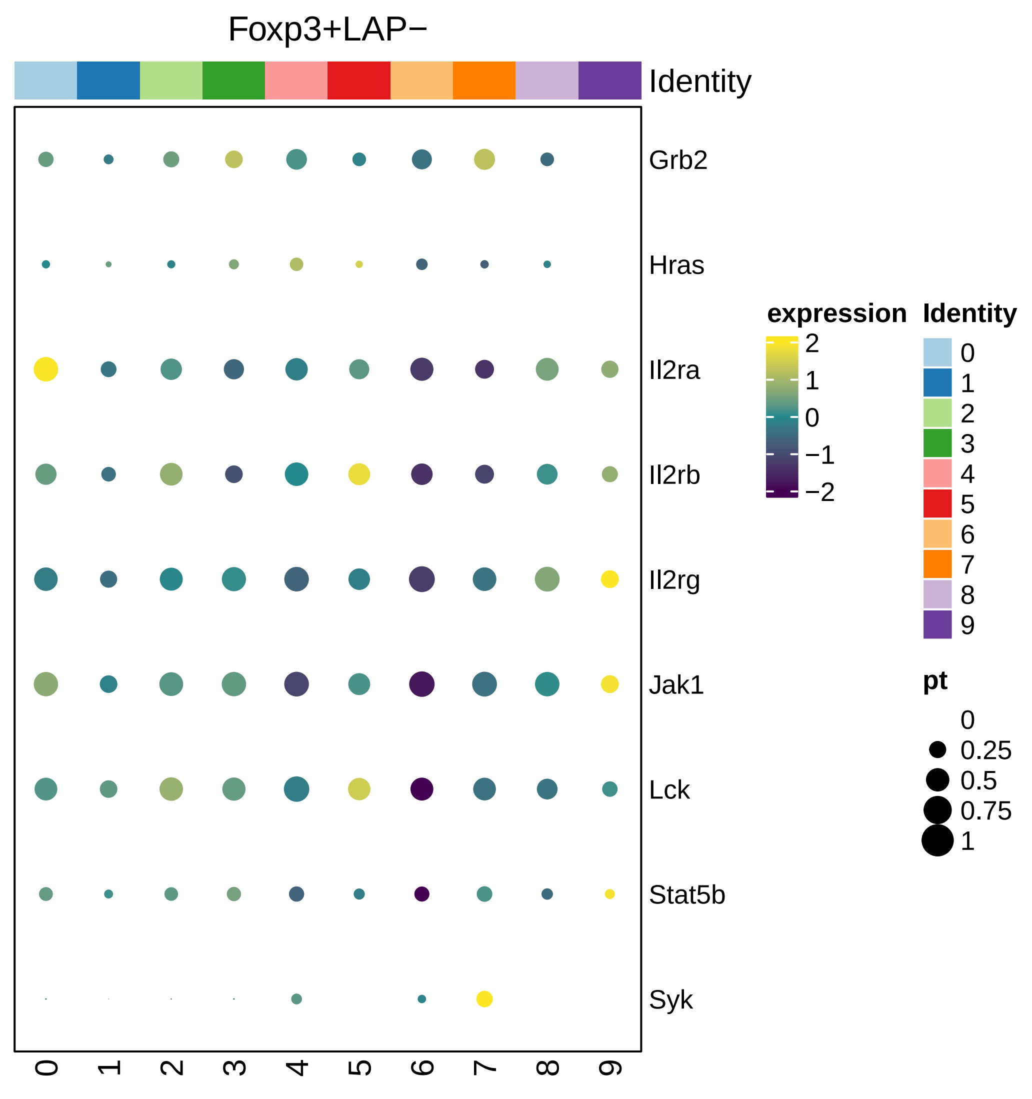

Foxp3+LAP−
Identity
Grb2
Hras
Il2ra
Il2rb
Il2rg
Jak1
Lck
Stat5b
Syk
0
1
2
3
4
5
6
7
8
9
expression
2
1
0
−1
−2
Identity
0
1
2
3
4
5
6
7
8
9
pt
0
0.25
0.5
0.75
1

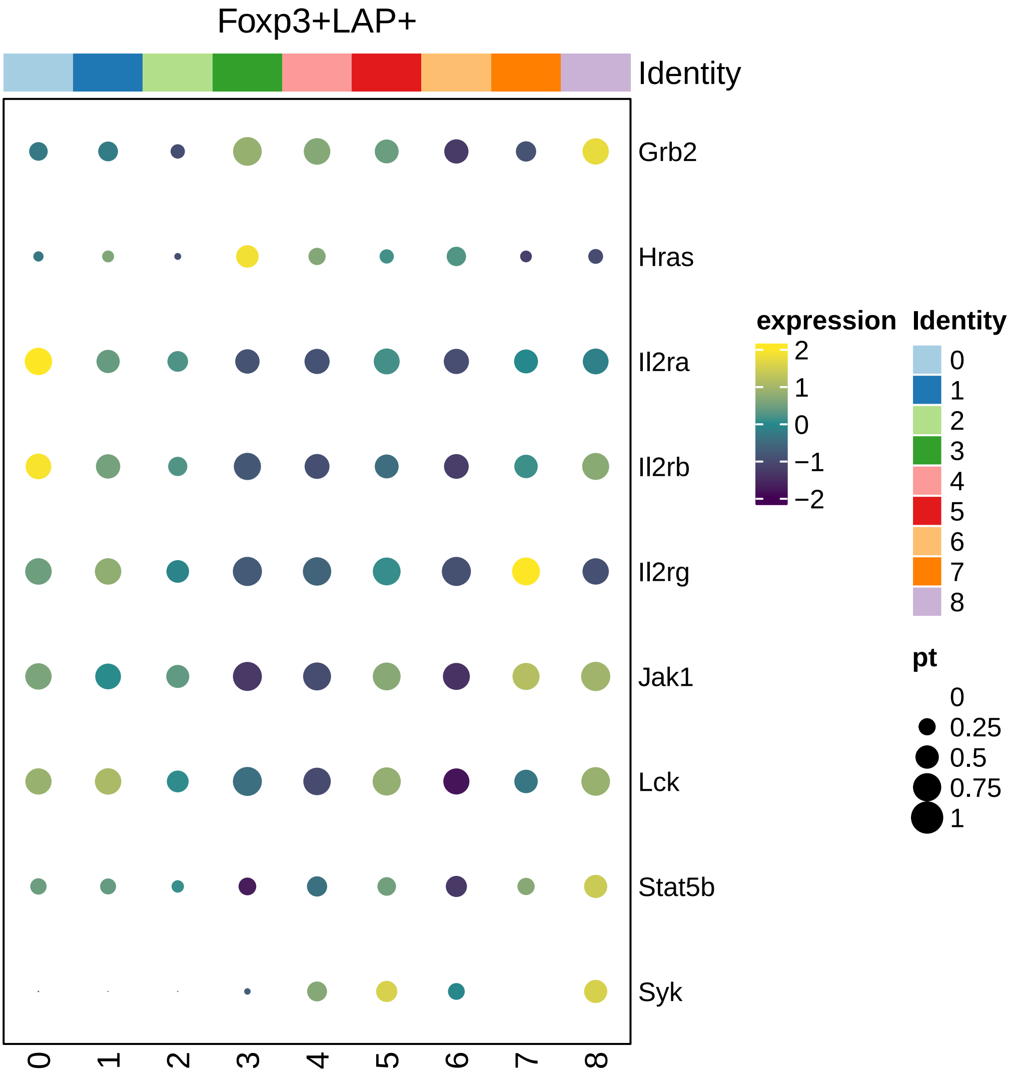

Foxp3+LAP+
Identity
Grb2
Hras
Il2ra
Il2rb
Il2rg
Jak1
Lck
Stat5b
Syk
0 1 2 3 4 5 6 7 8
expression
2
1
0
−1
−2
Identity
0
1
2
3
4
5
6
7
8
pt
0
0.25
0.5
0.75
1

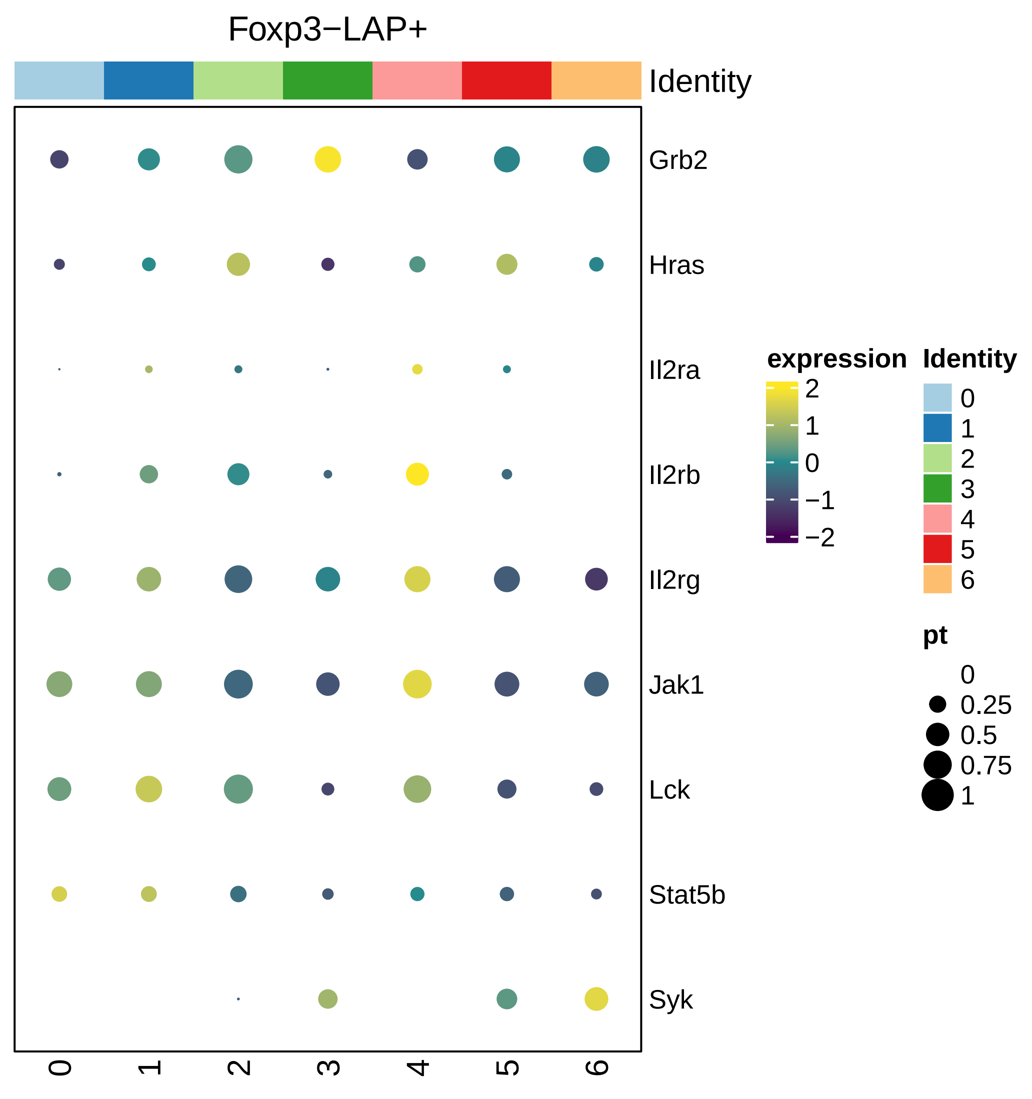
Foxp3−LAP+
Identity
Grb2
Hras
Il2ra
Il2rb
Il2rg
Jak1
Lck
Stat5b
Syk
0
1
2
3
4
5
6
expression
2
1
0
−1
−2
Identity
0
1
2
3
4
5
6
pt
0
0.25
0.5
0.75
1

IL-10 Clustered dot plot

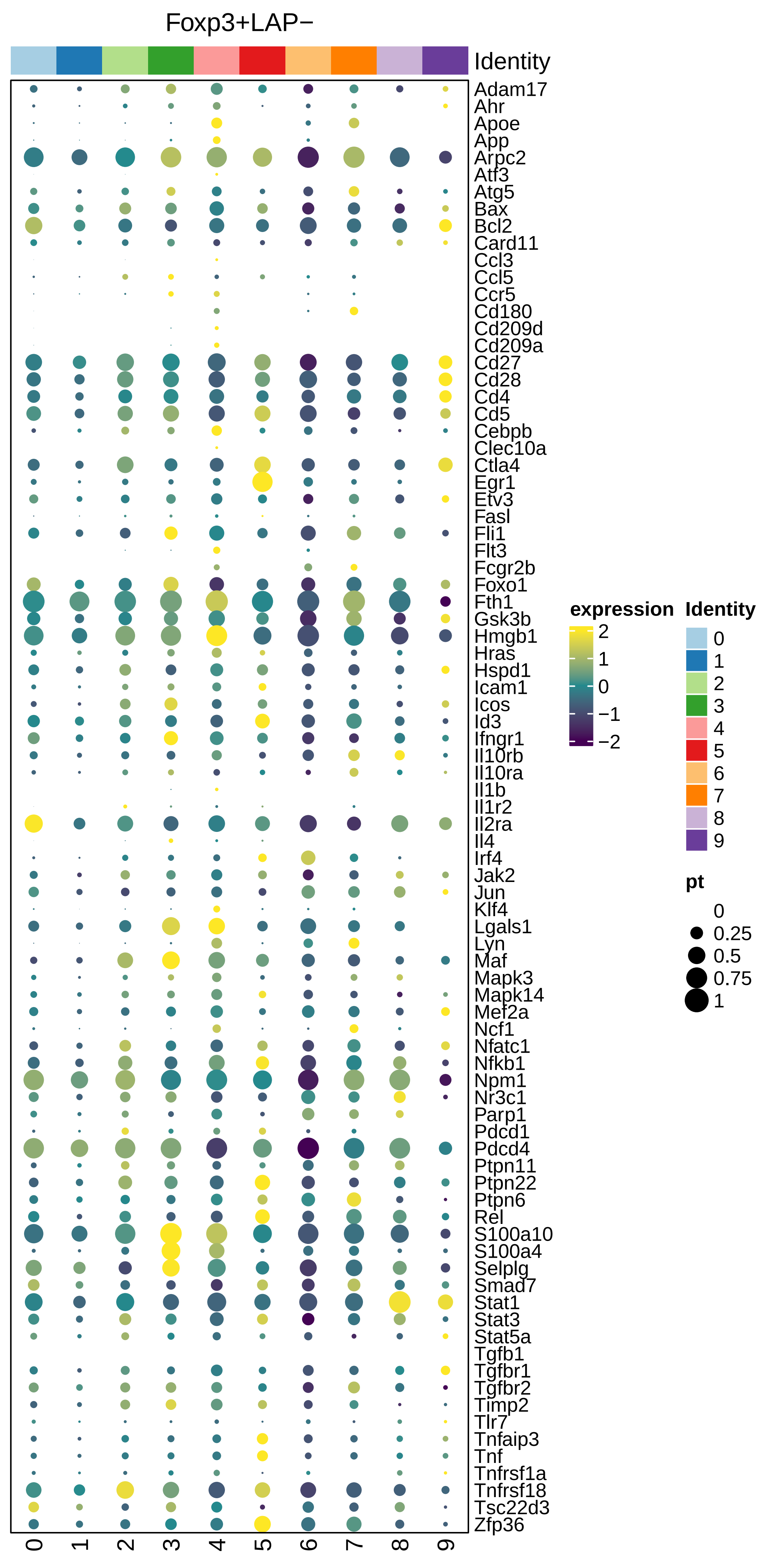
Foxp3+LAP−
Identity
Adam17
Ahr
Apoe
App
Arpc2
Atf3
Atg5
Bax
Bcl2
Card11
Ccl3
Ccl5
Ccr5
Cd180
Cd209d
Cd209a
Cd27
Cd28
Cd4
Cd5
Cebpb
Clec10a
Ctla4
Egr1
Etv3
Fasl
Fli1
Flt3
Fcgr2b
Foxo1
Fth1
Gsk3b
Hmgb1
Hras
Hspd1
Icam1
Icos
Id3
Ifngr1
Il10rb
Il10ra
Il1b
Il1r2
Il2ra
Il4
Irf4
Jak2
Jun
Klf4
Lgals1
Lyn
Maf
Mapk3
Mapk14
Mef2a
Ncf1
Nfatc1
Nfkb1
Npm1
Nr3c1
Parp1
Pdcd1
Pdcd4
Ptpn11
Ptpn22
Ptpn6
Rel
S100a10
S100a4
Selplg
Smad7
Stat1
Stat3
Stat5a
Tgfb1
Tgfbr1
Tgfbr2
Timp2
Tlr7
Tnfaip3
Tnf
Tnfrsf1a
Tnfrsf18
Tsc22d3
Zfp36
0
1
2
3
4
5
6
7
8
9
expression
2
1
0
−1
−2
Identity
0
1
2
3
4
5
6
7
8
9
pt
0
0.25
0.5
0.75
1

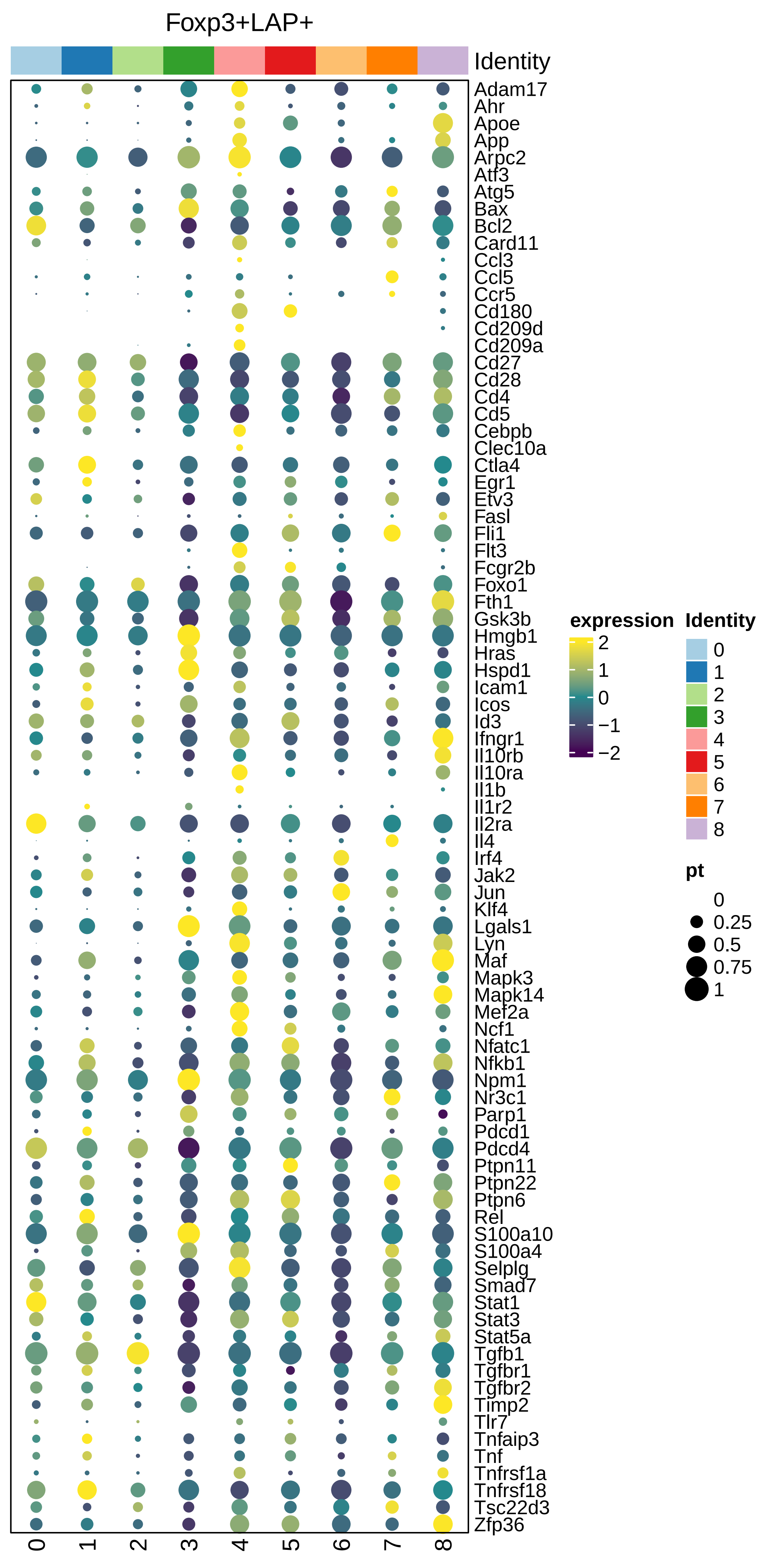
Foxp3+LAP+
Identity
Adam17
Ahr
Apoe
App
Arpc2
Atf3
Atg5
Bax
Bcl2
Card11
Ccl3
Ccl5
Ccr5
Cd180
Cd209d
Cd209a
Cd27
Cd28
Cd4
Cd5
Cebpb
Clec10a
Ctla4
Egr1
Etv3
Fasl
Fli1
Flt3
Fcgr2b
Foxo1
Fth1
Gsk3b
Hmgb1
Hras
Hspd1
Icam1
Icos
Id3
Ifngr1
Il10rb
Il10ra
Il1b
Il1r2
Il2ra
Il4
Irf4
Jak2
Jun
Klf4
Lgals1
Lyn
Maf
Mapk3
Mapk14
Mef2a
Ncf1
Nfatc1
Nfkb1
Npm1
Nr3c1
Parp1
Pdcd1
Pdcd4
Ptpn11
Ptpn22
Ptpn6
Rel
S100a10
S100a4
Selplg
Smad7
Stat1
Stat3
Stat5a
Tgfb1
Tgfbr1
Tgfbr2
Timp2
Tlr7
Tnfaip3
Tnf
Tnfrsf1a
Tnfrsf18
Tsc22d3
Zfp36
0
1
2
3
4
5
6
7
8
expression
2
1
0
−1
−2
Identity
0
1
2
3
4
5
6
7
8
pt
0
0.25
0.5
0.75
1

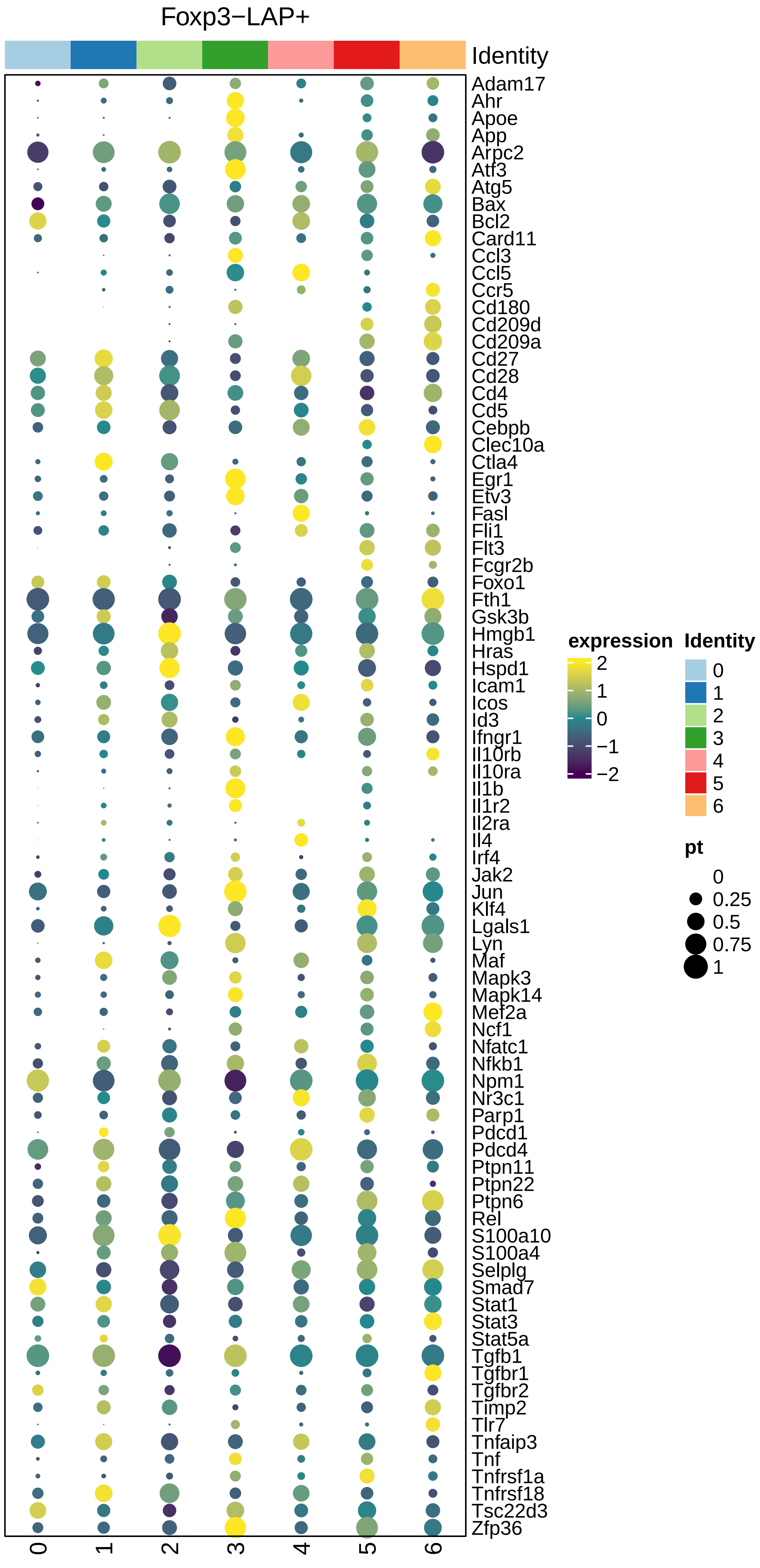

Foxp3−LAP+
Identity
Adam17
Ahr
Apoe
App
Arpc2
Atf3
Atg5
Bax
Bcl2
Card11
Ccl3
Ccl5
Ccr5
Cd180
Cd209d
Cd209a
Cd27
Cd28
Cd4
Cd5
Cebpb
Clec10a
Ctla4
Egr1
Etv3
Fasl
Fli1
Flt3
Fcgr2b
Foxo1
Fth1
Gsk3b
Hmgb1
Hras
Hspd1
Icam1
Icos
Id3
Ifngr1
Il10rb
Il10ra
Il1b
Il1r2
Il2ra
Il4
Irf4
Jak2
Jun
Klf4
Lgals1
Lyn
Maf
Mapk3
Mapk14
Mef2a
Ncf1
Nfatc1
Nfkb1
Npm1
Nr3c1
Parp1
Pdcd1
Pdcd4
Ptpn11
Ptpn22
Ptpn6
Rel
S100a10
S100a4
Selplg
Smad7
Stat1
Stat3
Stat5a
Tgfb1
Tgfbr1
Tgfbr2
Timp2
Tlr7
Tnfaip3
Tnf
Tnfrsf1a
Tnfrsf18
Tsc22d3
Zfp36
0
1
2
3
4
5
6
expression
2
1
0
−1
−2
Identity
0
1
2
3
4
5
6
pt
0
0.25
0.5
0.75
1

TGFβ Clustered dot plot

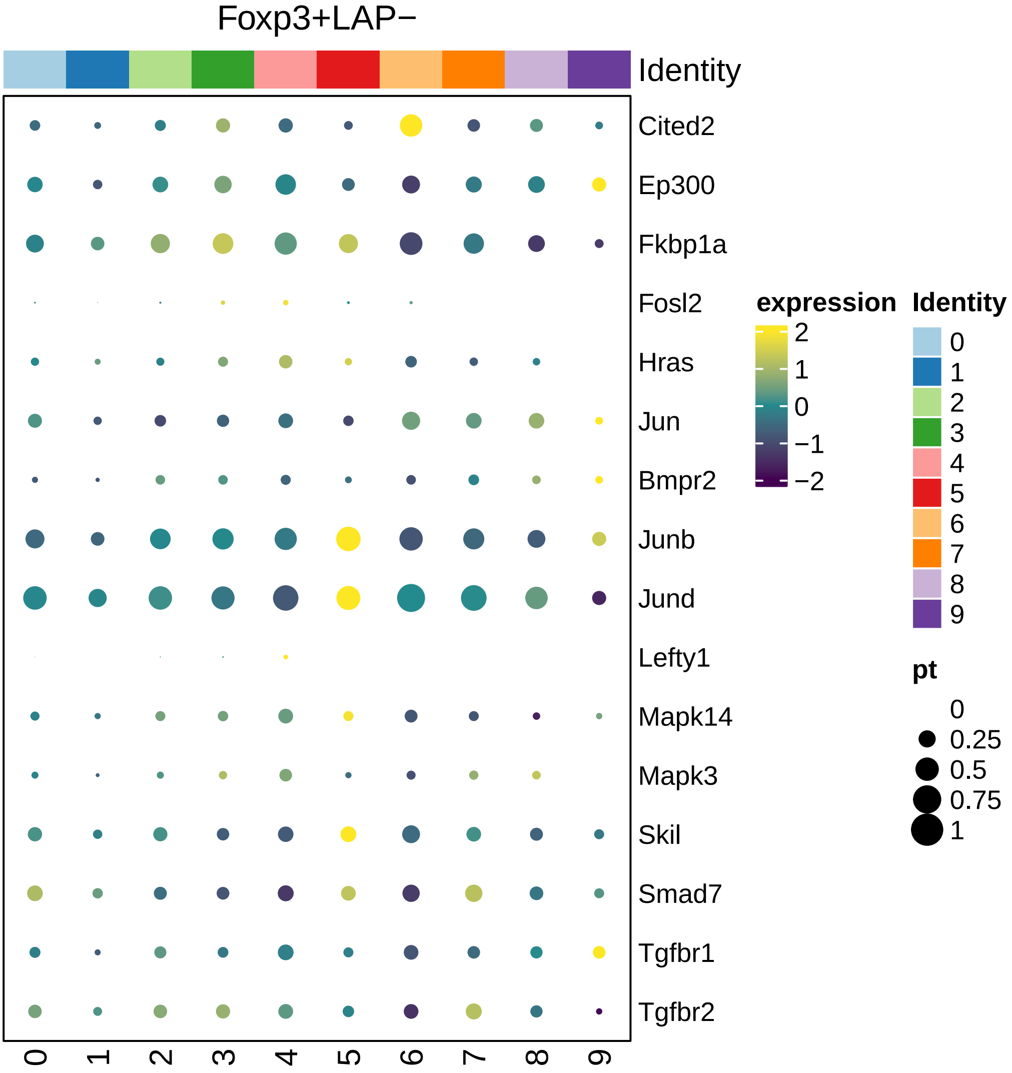
Foxp3+LAP−
Identity
Cited2
Ep300
Fkbp1a
Fosl2
Hras
Jun
Bmpr2
Junb
Jund
Lefty1
Mapk14
Mapk3
Skil
Smad7
Tgfbr1
Tgfbr2
0 1 2 3 4 5 6 7 8 9
expression
2
1
0
−1
−2
Identity
0
1
2
3
4
5
6
7
8
9
pt
0
0.25
0.5
0.75
1

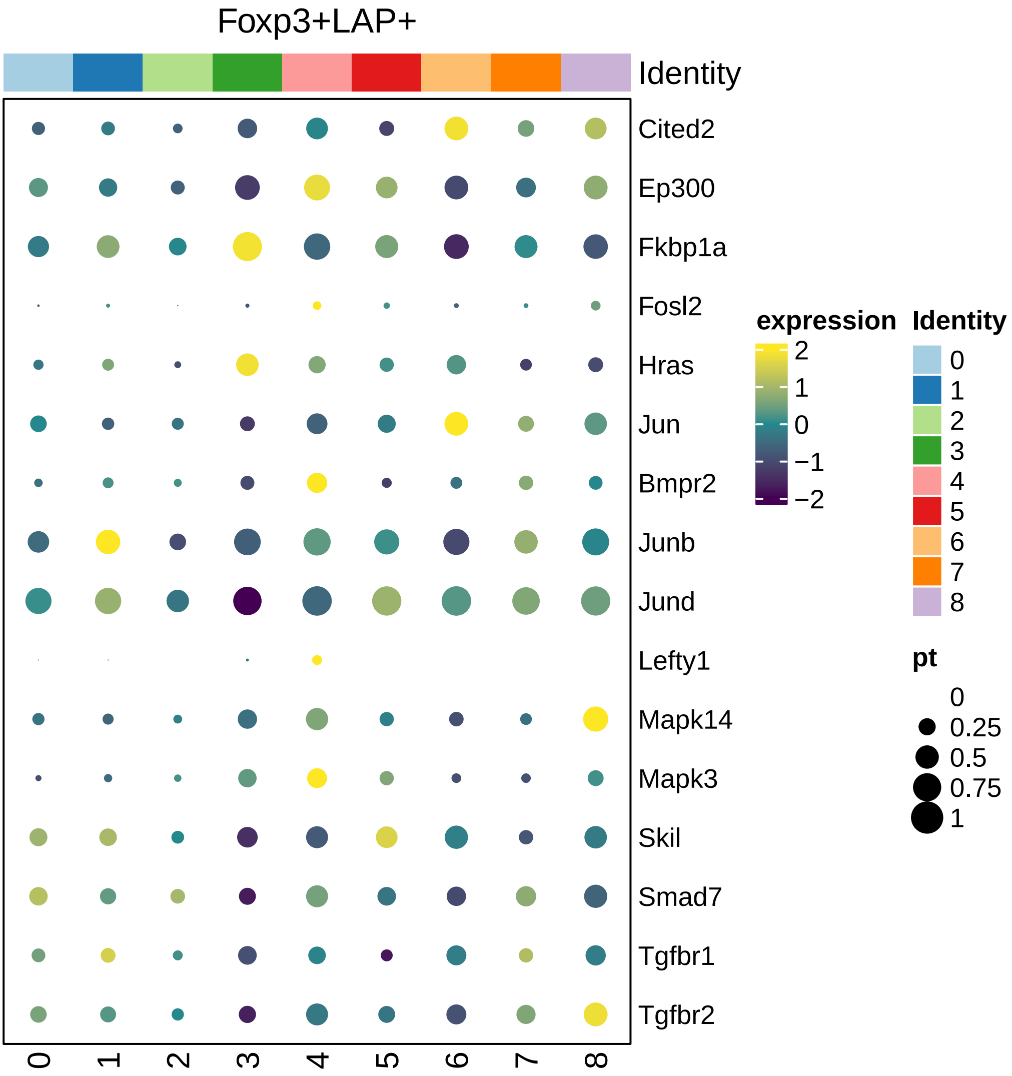
Foxp3+LAP+
Identity
Cited2
Ep300
Fkbp1a
Fosl2
Hras
Jun
Bmpr2
Junb
Jund
Lefty1
Mapk14
Mapk3
Skil
Smad7
Tgfbr1
Tgfbr2
0
1
2
3
4
5
6
7
8
expression
2
1
0
−1
−2
Identity
0
1
2
3
4
5
6
7
8
pt
0
0.25
0.5
0.75
1

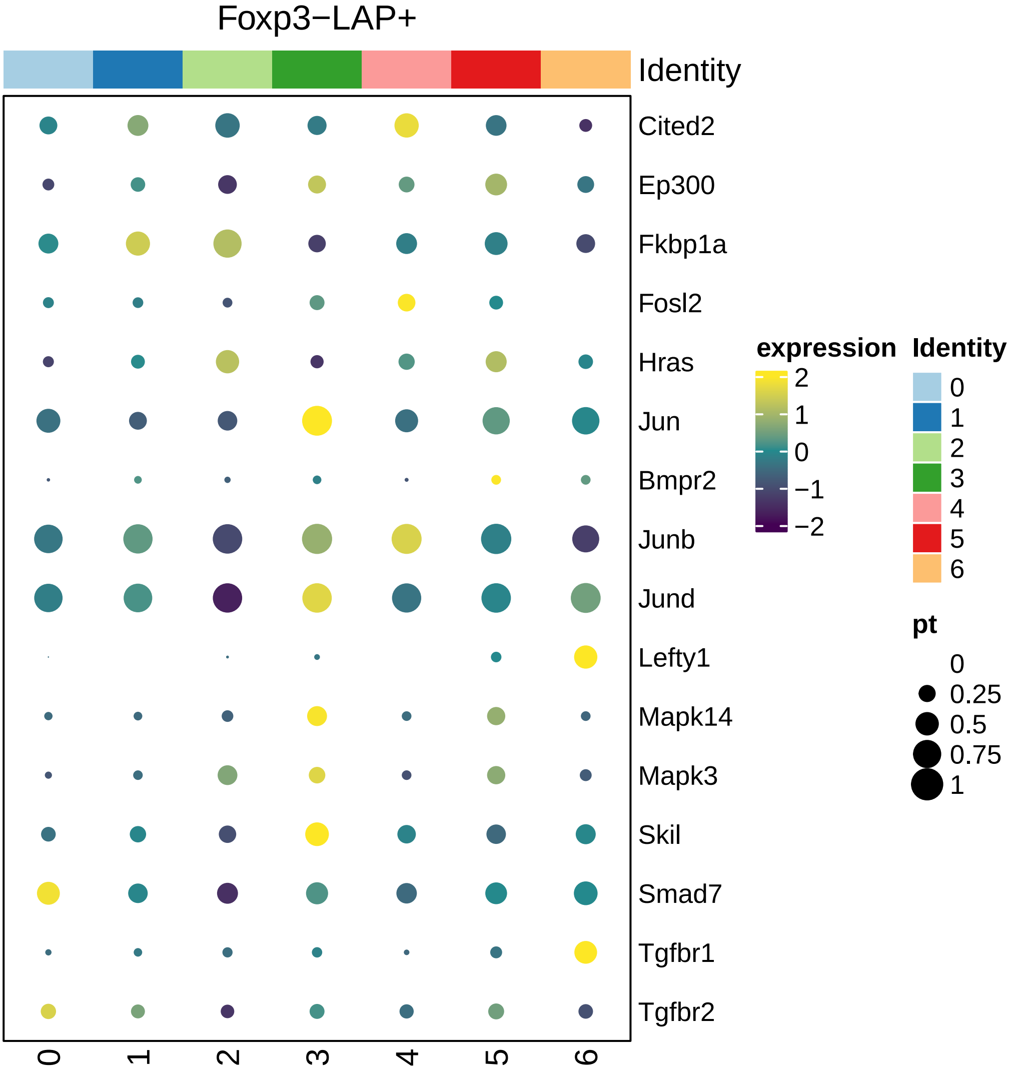
Foxp3−LAP+
Identity
Cited2
Ep300
Fkbp1a
Fosl2
Hras
Jun
Bmpr2
Junb
Jund
Lefty1
Mapk14
Mapk3
Skil
Smad7
Tgfbr1
Tgfbr2
0
1
2
3
4
5
6
expression
2
1
0
−1
−2
Identity
0
1
2
3
4
5
6
pt
0
0.25
0.5
0.75
1

TCR Clustered dot plot

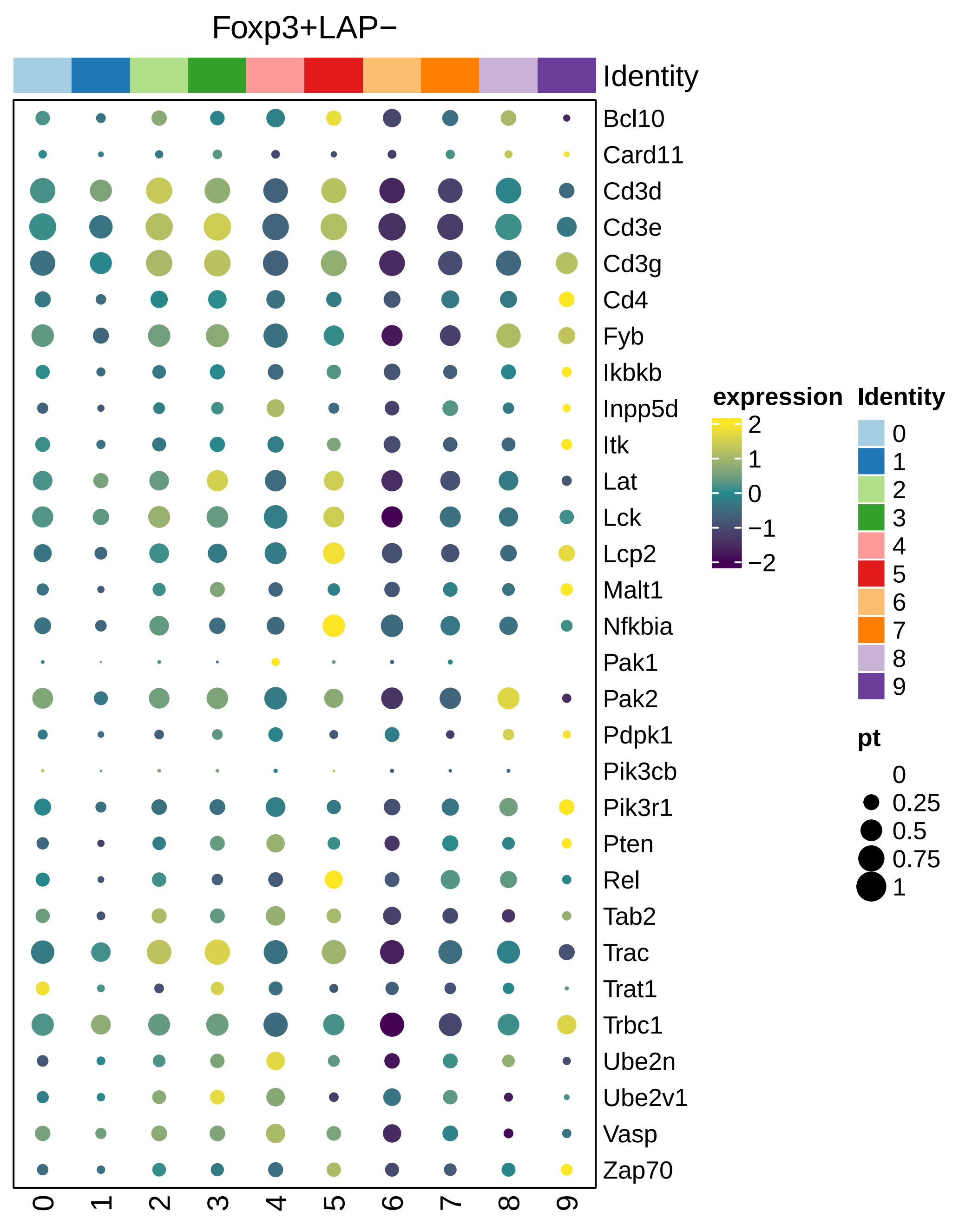
Foxp3+LAP−
Identity
Bcl10
Card11
Cd3d
Cd3e
Cd3g
Cd4
Fyb
Ikbkb
Inpp5d
Itk
Lat
Lck
Lcp2
Malt1
Nfkbia
Pak1
Pak2
Pdpk1
Pik3cb
Pik3r1
Pten
Rel
Tab2
Trac
Trat1
Trbc1
Ube2n
Ube2v1
Vasp
Zap70
0 1 2 3 4 5 6 7 8 9
expression
2 1 0 −1 −2
Identity
0 1 2 3 4 5 6 7 8 9
pt
0 0.25 0.5 0.75 1

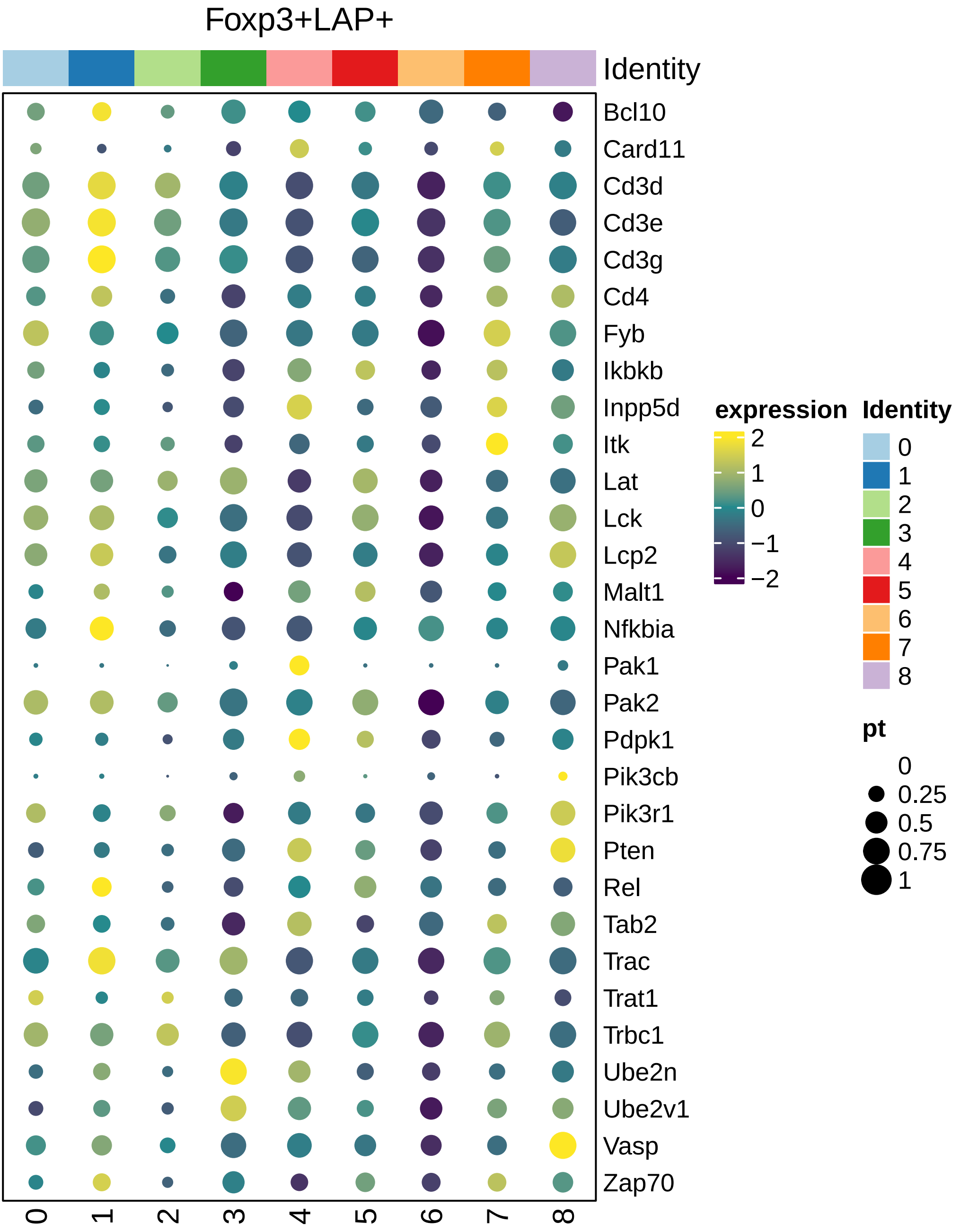

Foxp3+LAP+
Identity
Bcl10
Card11
Cd3d
Cd3e
Cd3g
Cd4
Fyb
Ikbkb
Inpp5d
Itk
Lat
Lck
Lcp2
Malt1
Nfkbia
Pak1
Pak2
Pdpk1
Pik3cb
Pik3r1
Pten
Rel
Tab2
Trac
Trat1
Trbc1
Ube2n
Ube2v1
Vasp
Zap70
0 1 2 3 4 5 6 7 8
expression
2
1
0
−1
−2
Identity
0
1
2
3
4
5
6
7
8
pt
0
0.25
0.5
0.75
1

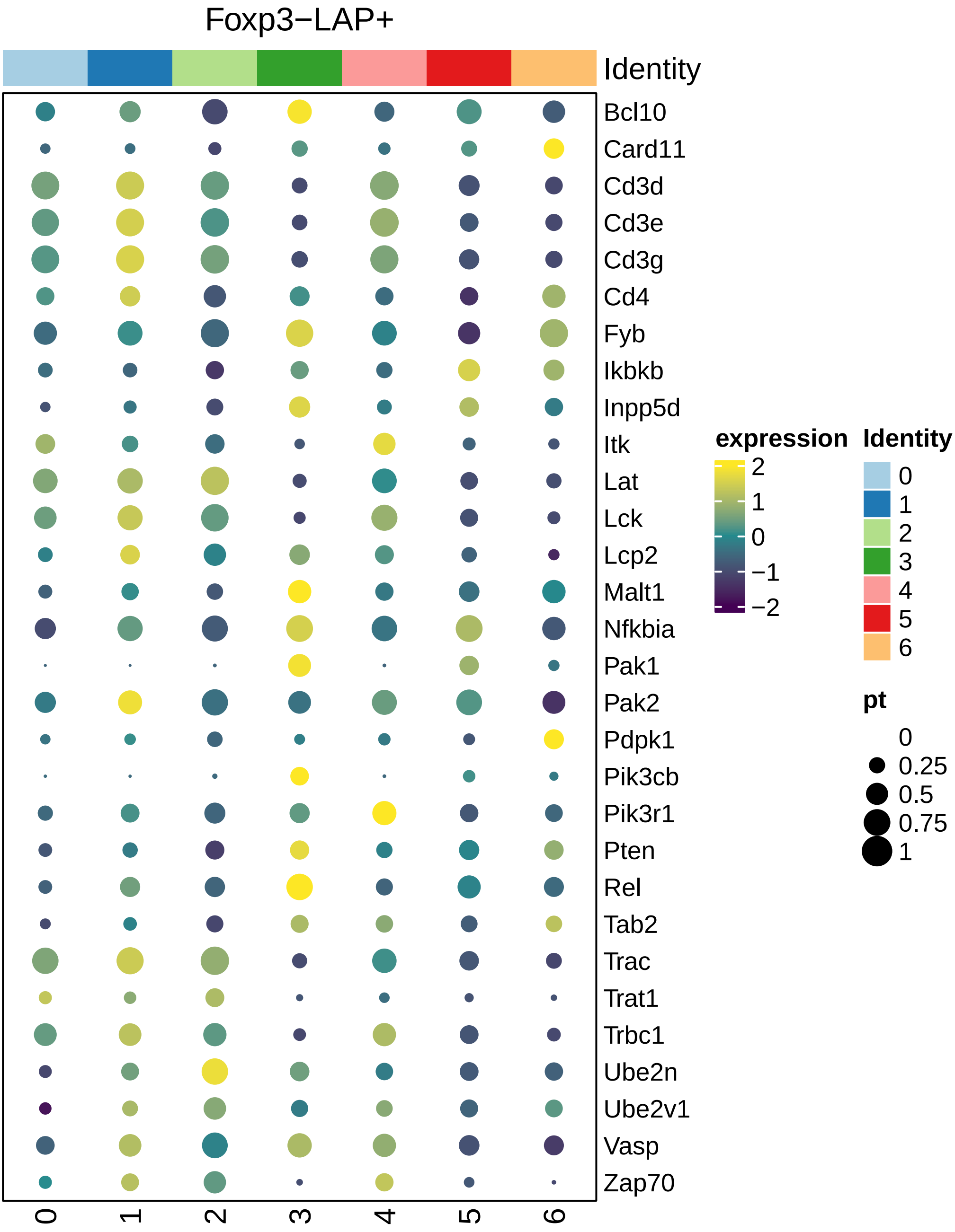
Foxp3−LAP+
Identity
Bcl10
Card11
Cd3d
Cd3e
Cd3g
Cd4
Fyb
Ikbkb
Inpp5d
Itk
Lat
Lck
Lcp2
Malt1
Nfkbia
Pak1
Pak2
Pdpk1
Pik3cb
Pik3r1
Pten
Rel
Tab2
Trac
Trat1
Trbc1
Ube2n
Ube2v1
Vasp
Zap70
0
1
2
3
4
5
6
expression
2
1
0
−1
−2
Identity
0
1
2
3
4
5
6
pt
0
0.25
0.5
0.75
1

MHCII Clustered dot plot

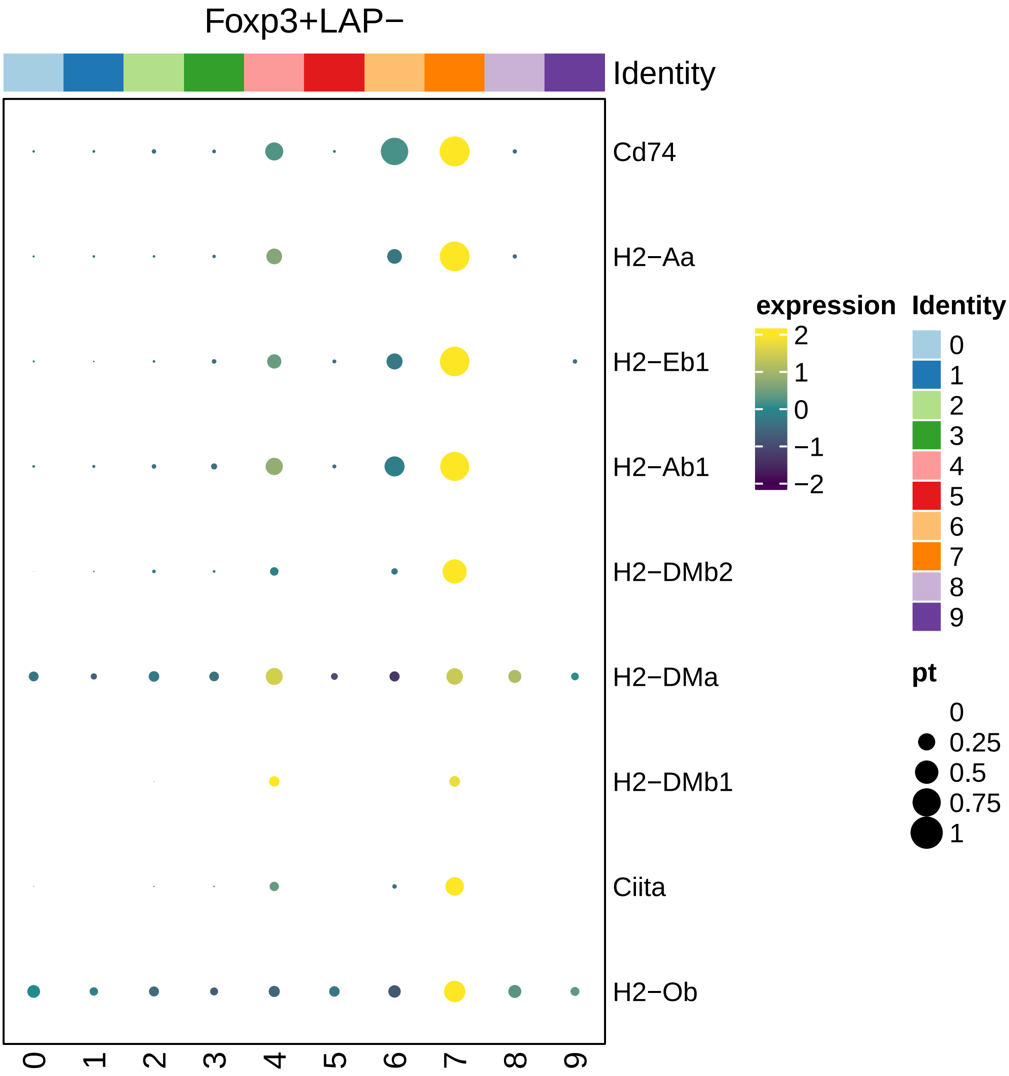
Foxp3+LAP−
Identity
Cd74
H2−Aa
H2−Eb1
H2−Ab1
H2−DMb2
H2−DMa
H2−DMb1
Ciita
H2−Ob
0
1
2
3
4
5
6
7
8
9
expression
2
1
0
−1
−2
Identity
0
1
2
3
4
5
6
7
8
9
pt
0
0.25
0.5
0.75
1

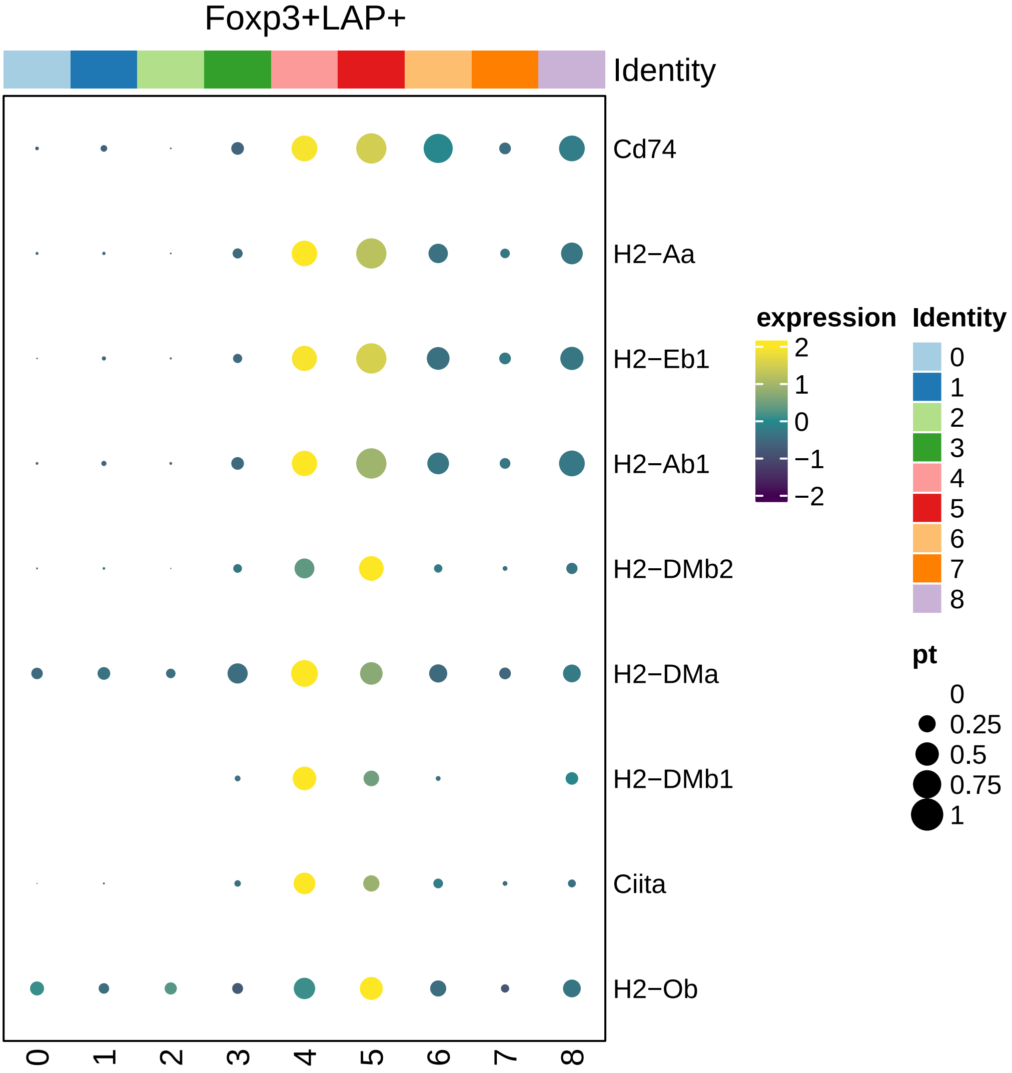

Foxp3+LAP+
Identity
Cd74
H2−Aa
H2−Eb1
H2−Ab1
H2−DMb2
H2−DMa
H2−DMb1
Ciita
H2−Ob
0
1
2
3
4
5
6
7
8
expression
2
1
0
−1
−2
Identity
0
1
2
3
4
5
6
7
8
pt
0
0.25
0.5
0.75
1

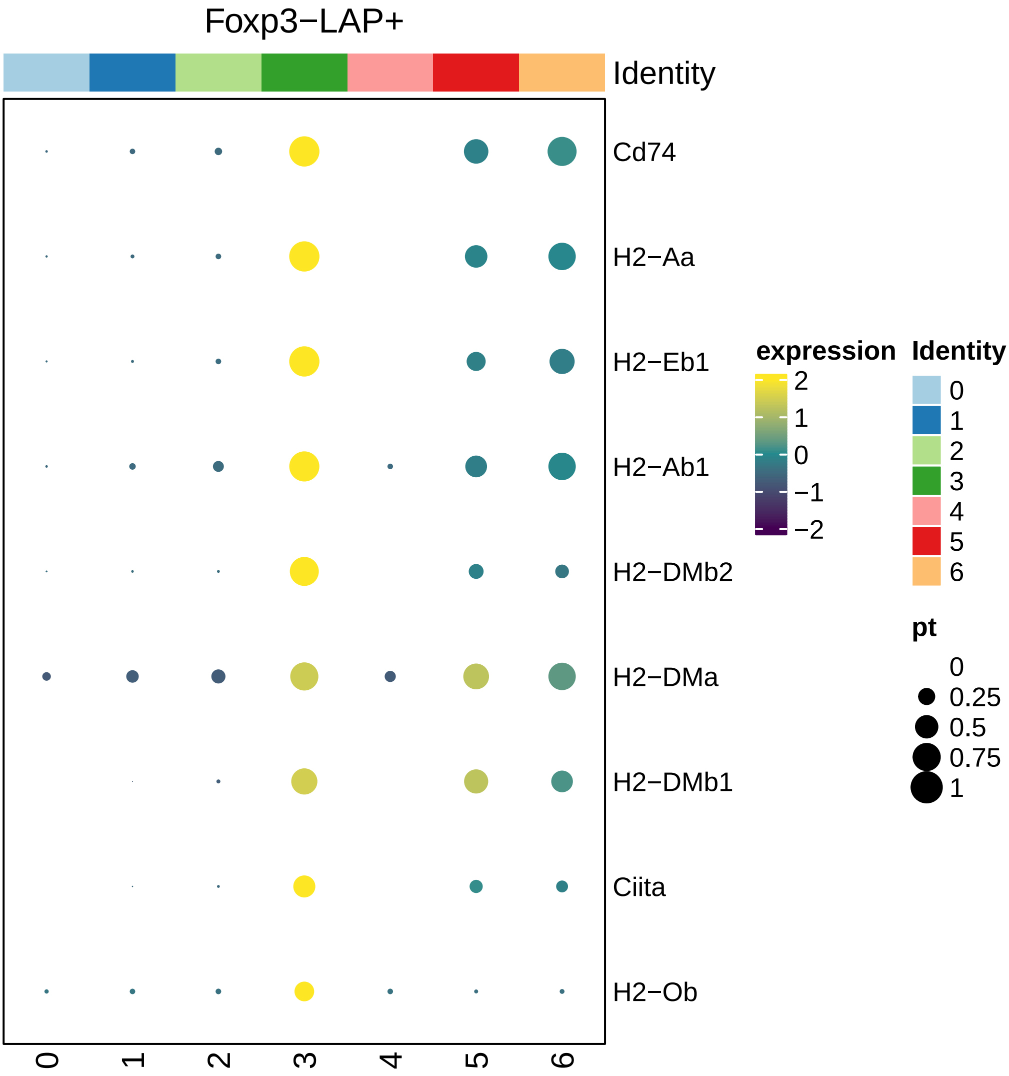
Foxp3−LAP+
Identity
Cd74
H2−Aa
H2−Eb1
H2−Ab1
H2−DMb2
H2−DMa
H2−DMb1
Ciita
H2−Ob
0
1
2
3
4
5
6
expression
2
1
0
−1
−2
Identity
0
1
2
3
4
5
6
pt
0
0.25
0.5
0.75
1

Myeloid Clustered dot plot

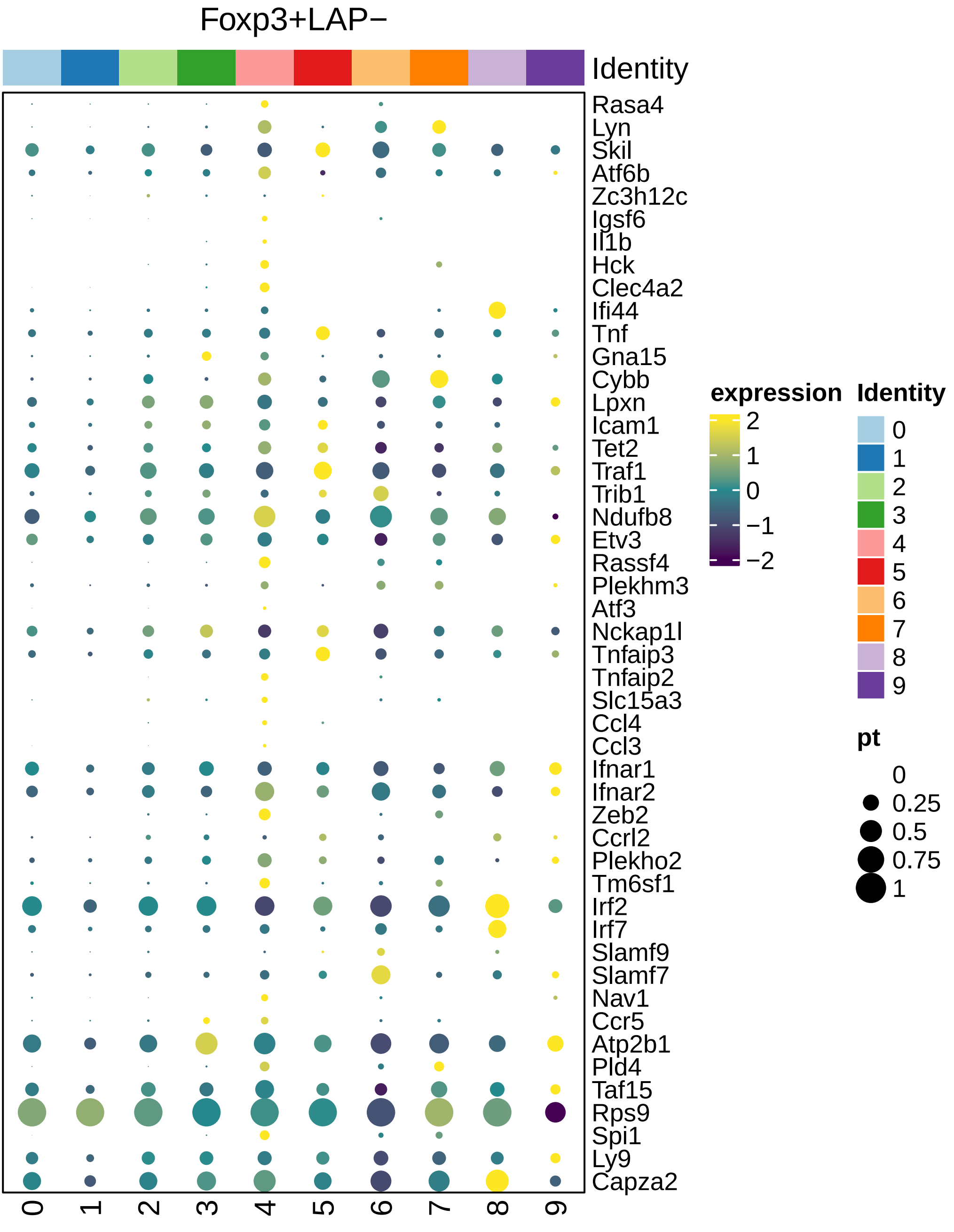

Foxp3+LAP−
Identity
Rasa4
Lyn
Skil
Atf6b
Zc3h12c
Igsf6
Il1b
Hck
Clec4a2
Ifi44
Tnf
Gna15
Cybb
Lpxn
Icam1
Tet2
Traf1
Trib1
Ndufb8
Etv3
Rassf4
Plekhm3
Atf3
Nckap1l
Tnfaip3
Tnfaip2
Slc15a3
Ccl4
Ccl3
Ifnar1
Ifnar2
Zeb2
Ccrl2
Plekho2
Tm6sf1
Irf2
Irf7
Slamf9
Slamf7
Nav1
Ccr5
Atp2b1
Pld4
Taf15
Rps9
Spi1
Ly9
Capza2
0 1 2 3 4 5 6 7 8 9
expression
2
1
0
−1
−2
Identity
0
1
2
3
4
5
6
7
8
9
pt
0
0.25
0.5
0.75
1

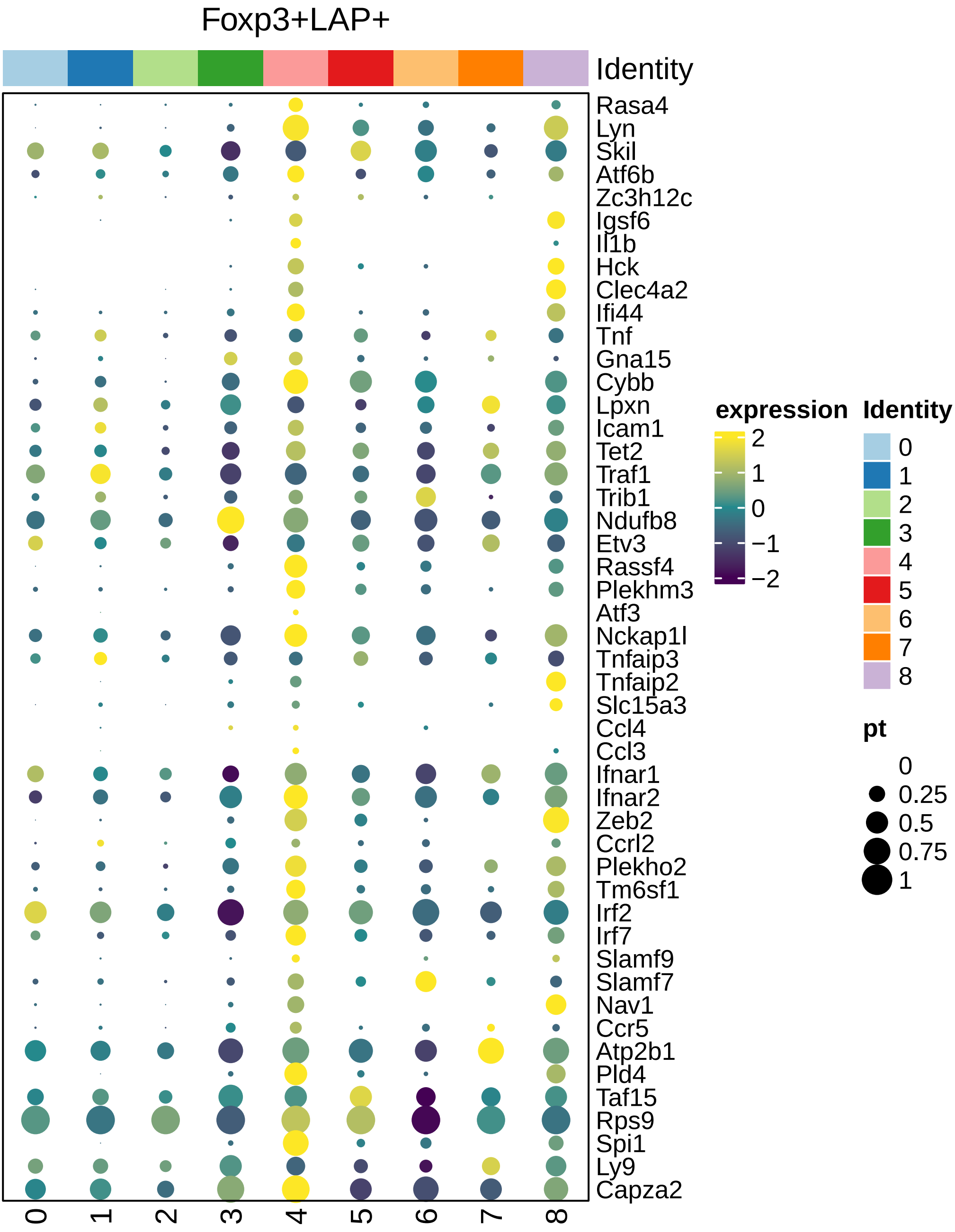

Foxp3+LAP+
Identity
Rasa4
Lyn
Skil
Atf6b
Zc3h12c
Igsf6
Il1b
Hck
Clec4a2
Ifi44
Tnf
Gna15
Cybb
Lpxn
Icam1
Tet2
Traf1
Trib1
Ndufb8
Etv3
Rassf4
Plekhm3
Atf3
Nckap1l
Tnfaip3
Tnfaip2
Slc15a3
Ccl4
Ccl3
Ifnar1
Ifnar2
Zeb2
Ccrl2
Plekho2
Tm6sf1
Irf2
Irf7
Slamf9
Slamf7
Nav1
Ccr5
Atp2b1
Pld4
Taf15
Rps9
Spi1
Ly9
Capza2
0
1
2
3
4
5
6
7
8
expression
2
1
0
−1
−2
Identity
0
1
2
3
4
5
6
7
8
pt
0
0.25
0.5
0.75
1

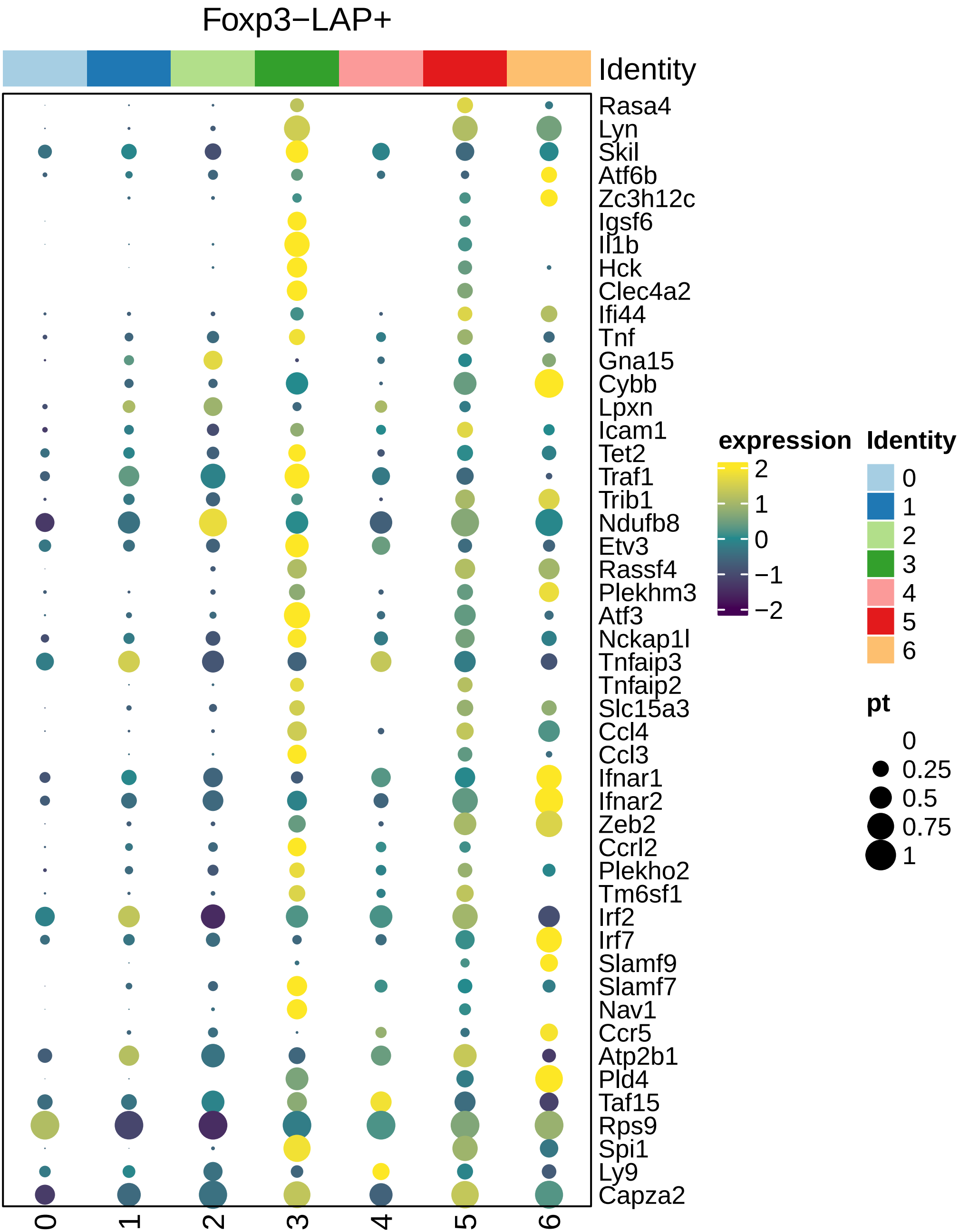

Foxp3−LAP+
Identity
Rasa4
Lyn
Skil
Atf6b
Zc3h12c
Igsf6
Il1b
Hck
Clec4a2
Ifi44
Tnf
Gna15
Cybb
Lpxn
Icam1
Tet2
Traf1
Trib1
Ndufb8
Etv3
Rassf4
Plekhm3
Atf3
Nckap1l
Tnfaip3
Tnfaip2
Slc15a3
Ccl4
Ccl3
Ifnar1
Ifnar2
Zeb2
Ccrl2
Plekho2
Tm6sf1
Irf2
Irf7
Slamf9
Slamf7
Nav1
Ccr5
Atp2b1
Pld4
Taf15
Rps9
Spi1
Ly9
Capza2
0
1
2
3
4
5
6
expression
2
1
0
−1
−2
Identity
0
1
2
3
4
5
6
pt
0
0.25
0.5
0.75
1
